# Light‐Switched Mesenchymal Stem Cells for In Situ Exosome Amplification in Craniofacial Bone Defect Reconstruction

**DOI:** 10.1002/advs.75519

**Published:** 2026-05-06

**Authors:** Tingting Wu, Yajing Liu, Shuman Wang, Xiaoming Bai, Luyun Zhang, Yuwei Liu, Zhiwen Fu, Chen Shi

**Affiliations:** ^1^ Department of Pharmacy, Union Hospital, Tongji Medical College Huazhong University of Science and Technology Wuhan Hubei China; ^2^ Hubei Province Clinical Research Center For Precision Medicine For Critical Illness Wuhan Hubei China; ^3^ Hubei Key Laboratory of Natural Active Polysaccharides, Union Hospital, Tongji Medical College Huazhong University of Science and Technology Wuhan Hubei China

**Keywords:** bone defect, exosome, mesenchymal stem cell, regenerative medicine, upconversion nanoparticles

## Abstract

Mesenchymal stem cell (MSC)‐based therapies hold great promise for tissue regeneration, yet precise spatiotemporal regulation of their bioactivity remains challenging. Here, we report a light‐switchable MSC system (MSC‐UCNPs) enabled by intracellular upconversion nanoparticles (UCNPs), which allowed remote control of exosome biogenesis and regenerative function. Upon 980 nm near‐infrared irradiation, intracellular UCNPs emitted localized 365 nm ultraviolet light without compromising MSC viability. The generated UVA stimulus activated the ROS/HEXB/LAMP1 signaling cascade, suppressing lysosome–multivesicular body fusion and thereby markedly enhancing exosome production (increased to 2.7‐fold). The MSC‐derived exosomes exerted autocrine effects to promote MSC proliferation and osteogenic differentiation, while also facilitating osteoblast maturation via activating the Wnt/β‐catenin pathway. To facilitate in vivo application, an injectable hydrogel composed of sodium alginate, calcium alginate, and hyaluronic acid was constructed through electrostatic interactions for the localized delivery of MSC‐UCNPs. Positron emission tomography–computed tomography (PET‐CT) imaging confirmed the in vivo light‐switchable behavior of MSC‐UCNPs, allowing on‐demand enhancement of in situ exosome release. Benefiting from the synergistic regenerative effects of MSCs and their exosomes, this light‐responsive MSC platform achieved robust cranial bone regeneration with a 3.2‐fold greater bone volume fraction compared to the control group.

## Graphical abstract

Light‐switchable MSCs (MSC‐UCNPs) were constructed by intracellular incorporation of UCNPs. Upon 980 nm irradiation, UCNPs emitted localized ultraviolet light (365 nm), activating the ROS/HEXB/LAMP1 signaling pathway to suppress lysosome–multivesicular body fusion and thereby enhance exosome biogenesis. Embedded within an injectable hydrogel, MSC‐UCNPs enable sustained retention and in situ exosome release, synergistically promoting osteogenic differentiation and craniofacial bone regeneration.

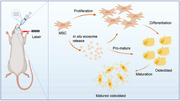

## 1 Introduction

Craniofacial defects are usually caused by trauma, tumors, infections, or congenital developmental defects, and seriously affect the quality of life and long‐term prognosis. Bone grafting remains the primary clinical intervention for defect repair. Autologous bone grafting, despite its osteogenic superiority, is constrained by limited donor availability, secondary surgical trauma, and donor‐site morbidity. Allogeneic bone transplantation is further hindered by immune rejection, infection risk, disease transmission, and insufficient biological activity [1]. Synthetic bone substitutes, such as bioceramics and bioglasses, exhibit favorable osteoconductivity but suffer from inadequate osteoinductivity and suboptimal biomechanical performance [2]. Other treatments, such as low‐frequency ultrasound and mechanical stimulation mediated by pulsed electromagnetic fields, are non‐invasive and have low side effects, but their effects are poor, and they are usually only used as adjuvant treatments [3]. Therefore, the treatment of craniofacial bone defects still faces great challenges.

Mesenchymal stem cells (MSCs), characterized by self‐renewal capacity and multilineage differentiation potential, have emerged as a promising cell source for bone regeneration [4]. As the primary progenitors of osteoblasts in vivo, MSCs play a central role in bone homeostasis and repair [5]. MSC‐based therapies have attracted increasing attention as a biologically active alternative to conventional grafting strategies. Nevertheless, their clinical translation is impeded by poor in vivo survival, phenotypic heterogeneity, and unpredictable therapeutic outcomes [6]. Exosomes, as a novel “cell‐free therapy”, have shown great application potential in the diagnosis and treatment of tissue repair, chronic diseases, autoimmune diseases, and tumors [7]. Exosomes carry a diverse repertoire of bioactive molecules such as proteins, nucleic acids, lipids, and small molecules from the donor cells, thereby recapitulating many of the paracrine functions of their parent cells. In addition, exosomes have a phospholipid bilayer structure, which cannot only protect the bioactive molecules but also transfer them from parent cells to recipient cells, effectively mediating the great challenges biological communication between cells [8]. However, the application of exosomes is greatly hampered by low production yields, high purification costs, and rapid clearance by the mononuclear phagocyte system following systemic administration, resulting in limited accumulation at target sites [9].

Whether MSCs or MSC‐derived exosomes (MSC‐Exos) are administered directly, conventional strategies essentially deliver a finite dose of therapeutic agents to the defect site. This paradigm inherently limits therapeutic durability and spatiotemporal control. An ideal regenerative strategy would enable MSCs to continuously generate exosomes in situ, effectively functioning as a localized living biopharmaceutical factory. On the one hand, MSCs not only serve as a renewable source of exosomes but also actively participate in tissue regeneration through osteogenic differentiation and the secretion of cytokines and growth factors [10]. On the other hand, locally produced exosomes can achieve high effective concentrations at the defect site, circumventing the need for complex isolation procedures while minimizing systemic loss and off‐target effects. Meanwhile, exosomes, as intracellular signaling messengers, can not only act on the parental MSCs to promote their osteogenic differentiation [11], but also act on osteoblasts to promote their maturation [12, 13]. Integrating sustained cellular activity with localized exosome‐mediated signaling thus represents a compelling and underexplored strategy for bone regeneration.

Among various biological materials derived from natural and synthetic sources, hydrogels have emerged as versatile platforms for the effective delivery of cells, bioactive factors, and therapeutic agents, owing to their high permeability, exceptional water retention capacity, and excellent biocompatibility [14]. In recent years, progress has been made in the development of multifunctional hydrogel systems for tissue regeneration. For instance, Ye et al. developed a bilayer hydrogel dressing for the comprehensive management of diabetic ulcer wounds. This system utilizes light‐triggered sequential release of nanosheets and nitric oxide to promote the repair of methicillin‐resistant Staphylococcus aureus (MRSA)‐infected wounds [15]. In parallel, Xia et al. designed another innovative biodegradable hydrogel platform comprising salmon skin‐derived extracellular matrix functionalized with transition metal oxides, exerting dual antibacterial and tissue‐repairing effects in MRSA‐infected wounds [16].

**SCHEME 1:**
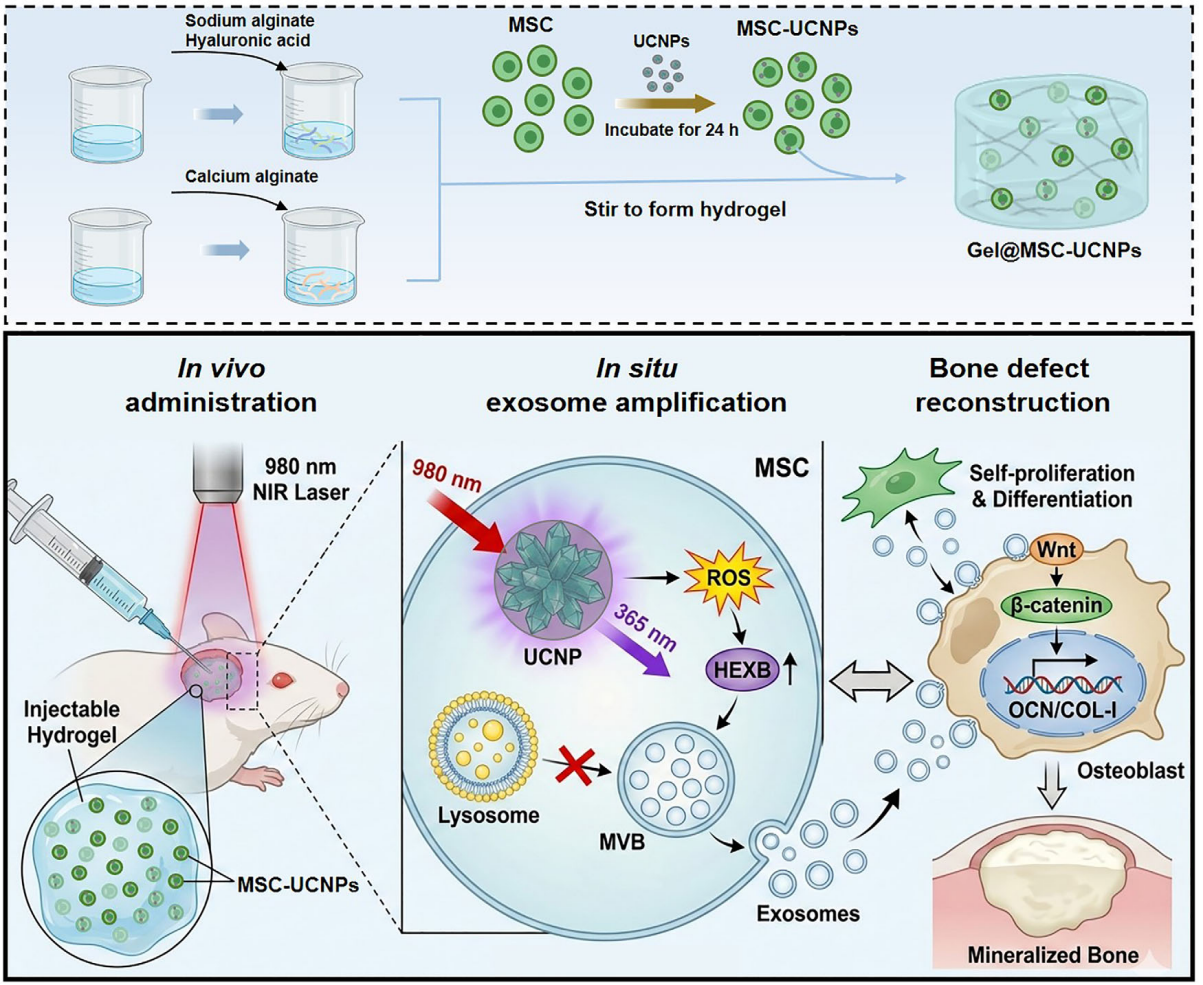
Schematic illustration of the preparation of Gel@MSC‐UCNPs and their role in craniofacial bone defect reconstruction.

In this work, we developed a kind of lightswitchable MSC platform for in situ exosome generation and craniofacial bone defect repair. Bone marrow–derived MSCs were readily internalized with upconversion nanoparticles (UCNPs, NaYF_4_: 20%Yb, 0.5%Tm) through a simple co‐incubation process, yielding MSC‐UCNPs. MSC‐UCNPs were further embedded in an injectable hydrogel (Gel@MSC‐UCNPs) for administration in vivo. Upon 980 nm near‐infrared irradiation, intracellular UCNPs generated ultraviolet emission, triggering enhanced exosome release from MSCs in situ. By synergistically integrating the regenerative capacity of MSCs with the paracrine potency of MSC‐derived exosomes, this light‐responsive system markedly accelerated craniofacial bone defect repair (Scheme 1).

## 2 Results

### 2.1 UVA Irradiation Promotes Exosome Release From MSCs

**FIGURE 1:**
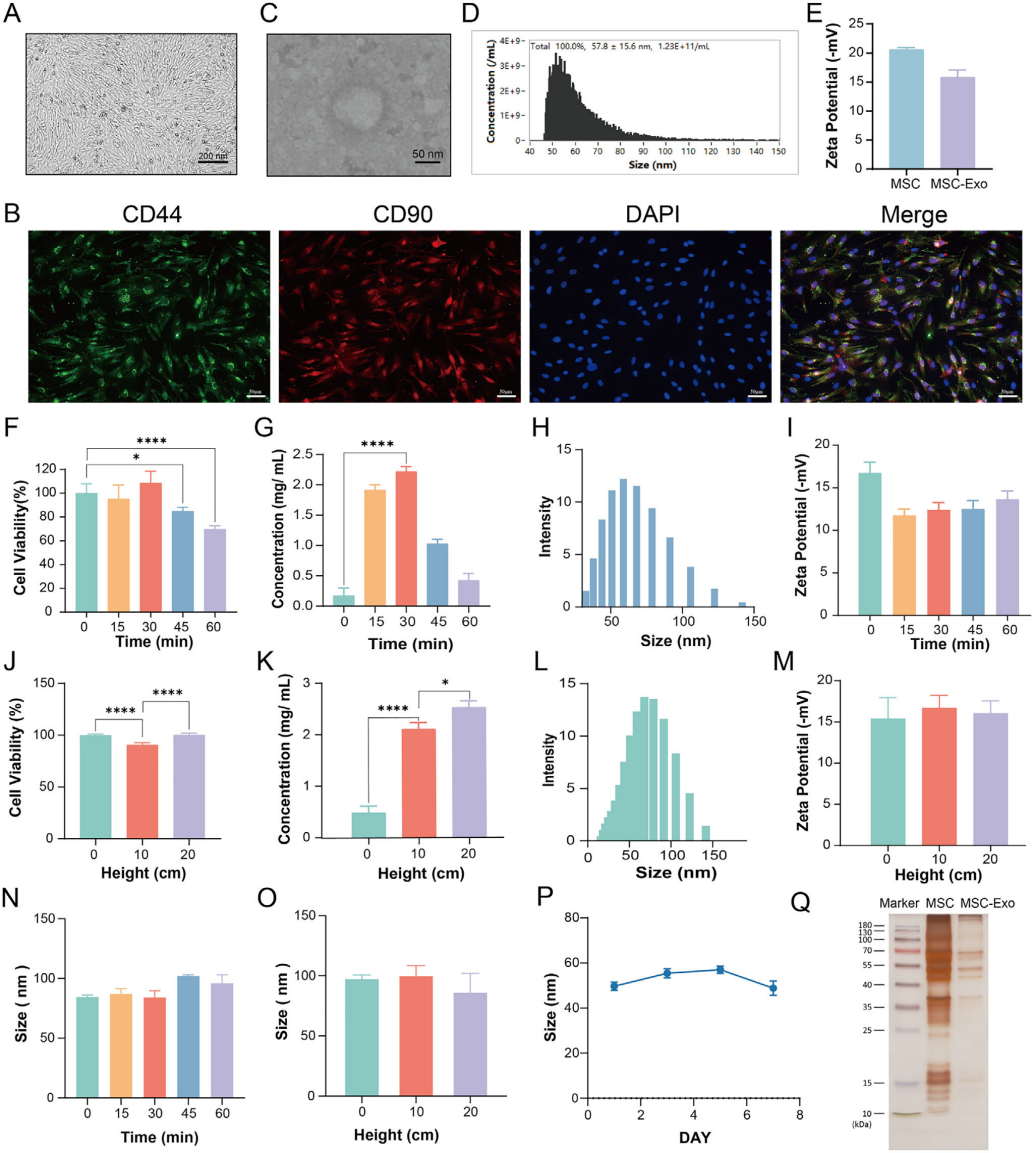
UVA promoted the release of MSC‐Exo in vitro. (A) Micrograph of MSCs morphology (Scale bar: 200 µm). (B) Immunofluorescence identification of MSCs (Scale bar: 50 µm). (C) TEM image of MSC‐Exo (Scale bar: 50 nm). (D) Size distribution profile of isolated MSC‐Exo. (E) Zeta potential of MSCs and MSC‐Exo (*n* = 3). (F) Cell viability of MSCs induced by UVA with different irradiation durations (Irradiation height: 20 cm) (*n* = 4), *p < 0.05, ****p < 0.0001. (G–I) Statistics of exosomal protein quantity, hydrodynamic diameter, and zeta potential released by MSCs exposed to UVA at different durations (Irradiation height: 20 cm) (*n* = 3), ****p < 0.0001. (J) Cell viability of MSCs induced by UVA with different irradiation heights (Irradiation duration: 30 min) (*n* = 4), ****p < 0.0001. (K–M) Statistics of exosomal protein quantity, hydrodynamic diameter, and zeta potential released by MSCs exposed to UVA at different heights (Irradiation duration: 30 min) (*n* = 3), *p < 0.05, ****p < 0.0001. (N) The hydrated particle sizes of exosomes at different time points. (O) The hydrated particle sizes of exosomes at different irradiation heights. (P) The stability of exosomes under the optimal irradiation conditions (irradiation time: 30 min, irradiation height: 20 cm). (Q) SDS‐PAGE image of MSC and MSC‐Exo.

MSCs were isolated from the femora and tibiae of Sprague‐Dawley (SD) rats and cultured in DMEM/F12 complete medium for 9 days. The cells adhered to the culture flask and exhibited a typical long spindle‐shaped morphology (Figure 1A). The identity of MSCs was confirmed by immunofluorescence staining. Previous studies have demonstrated that MSCs express surface markers including CD44, CD73, CD90, and CD105 [17]. The co‐localization of CD44 and CD90 confirmed the MSC phenotype (Figure 1B). Exosomes were isolated from MSC supernatant. Transmission electron microscopy (TEM) revealed that exosomes displayed a cup‐shaped morphology with a diameter predominantly ranging from 30 to 150 nm. The MSC‐Exo exhibited a zeta potential of −15.83 ± 3.1 mV (Figure 1C–E). These features were consistent with the typical characteristics of exosomes.

It is well established that exosome secretion is regulated by multiple factors, including cell type, microenvironmental conditions, and external stress stimuli [18, 19]. Previous studies have demonstrated that UVA‐based phototherapy can significantly enhance extracellular vesicle production [20]. Hence, the impact of UVA irradiation (wavelength: 365 nm) on exosome secretion was first investigated. It was found that exposure to 365 nm ultraviolet light for less than 30 min showed good biosafety toward MSCs (Figure 1F). Regarding irradiation distance, a distance of less than 20 cm significantly reduced MSC viability (Figure 1J). Herein, the irradiation height was set at 20 cm. With the extension of irradiation time, the amount of exosomes secreted gradually increased and reached a peak at 30 min, which was therefore chosen as 30 min as the irradiation time (Figure 1G–I). Moreover, by adjusting the irradiation time, it was observed that MSCs secreted the greatest amount of exosomes when the irradiation height was 20 cm (Figure 1K–M). The hydration particle sizes of exosomes at different times and at different heights as well as the optimal conditions (with an irradiation duration of 30 min and an irradiation height of 20 cm), were measured. All of these exosomes showed stability during the measurement period (Figure 1N–P). In addition, SDS‑PAGE analysis was performed to study the protein component of MSCs and MSC‑derived exosomes (MSC‑Exo). The results indicated that MSC‑Exo exhibited similar protein profiles to the parental MSCs (Figure 1Q). These findings provide a reference for subsequent studies on near‐infrared light stimulation of exosome secretion.

### 2.2 Light‐Switched MSC‐UCNPs Promoted the Release of Exosomes via ROS/ HEXB/ LAMP1 Pathway

**FIGURE 2:**
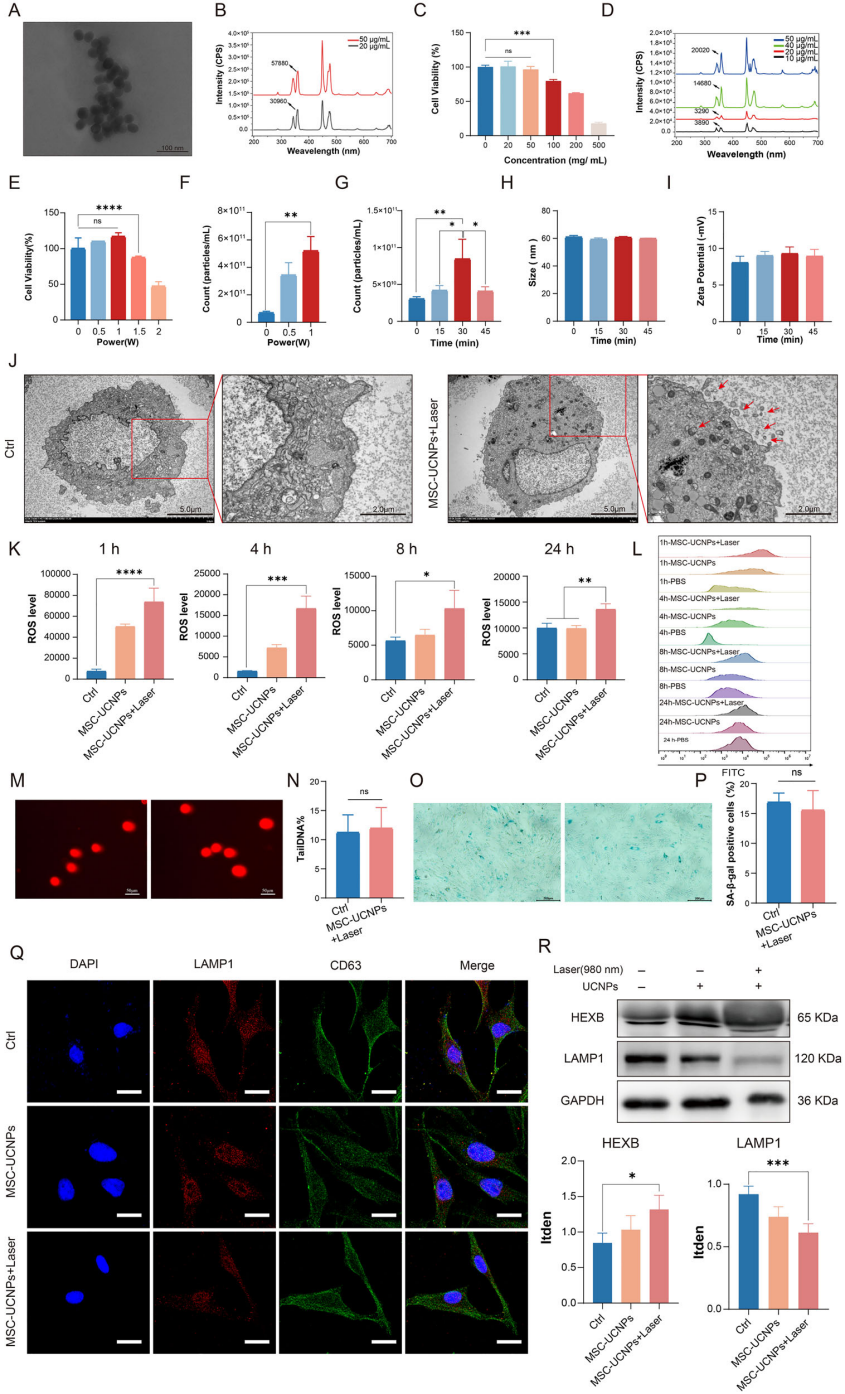
Light‐switched UCNPs promoted the release of exosomes from MSCs. (A) The morphology of UCNPs observed by TEM (Scale bar, 100 µm). (B) Excitation spectra of different concentrations of UCNPs under 980 nm (NIR) excitation light were obtained by using a time‐resolved fluorescence spectrometer. (C) Evaluation of the cell compatibility of UCNPs with MSCs in vitro (*n* = 3), ***p < 0.001. (D) The emission spectra of MSC‐UCNPs under 980 nm. (E) Evaluation of cellular damage to MSCs induced by 980 nm lasers of different power densities in vitro (*n* = 3), ***p < 0.001. (F) The statistical data of exosome particles released by MSC‐UCNPs under 980 nm laser irradiation at powers of 0, 0.5, and 1 W for 30 min were obtained via NTA analysis (*n* = 3), **p < 0.001. (G) The statistical data of exosome particles released by MSC‐UCNPs under 980 nm irradiation at 0, 15, 30, and 45 min were obtained by NTA analysis (*n* = 3), *p < 0.05, **p < 0.001. (H) The hydration particle size analysis of MSC‐UCNPs‐Exo obtained from different treatment groups (*n* = 3). (I) The zeta potential analysis of MSC‐UCNPs‐Exo obtained from different treatment groups (*n* = 3). (J) The biological process of exosome release by MSC‐UCNPs was observed by TEM. Left: the control group; right: the MSC‐UCNPs + Laser group, red arrow: EVs. (K) The oxidative stress analysis of MSCs was conducted at 1, 4, 8, and 24 h after NIR treatment (*n* = 3). The MSCs were divided into three groups: the control group, the MSC‐UCNPs group, and the MSC‐UCNPs + Laser group, *p < 0.05, **p < 0.001, ***p < 0.001, ****p < 0.0001. (L) The images of flow cytometry analysis after NIR treatment at 1, 4, 8, and 24 h. (M) Comet assay was performed to evaluate DNA damage. The cells were divided into two groups: the control group (Ctrl) and MSC‑UCNPs + laser irradiation group. (N) Statistical analysis of comet assay data. (O) Cellular senescence was determined by senescence‐associated β‐galactosidase (SA‐β‐gal) staining. (P) Quantification and statistical analysis of senescent cell proportion. (Q) Colocalization of MVB and lysosome was determined by immunofluorescence assays. (R) Representative expression of HEXB and LAMP1 by western blot. The quantitative analysis of HEXB and LAMP1 normalized to GAPDH (*n* = 3), *p < 0.05, ***p < 0.001.

Direct ultraviolet A (UVA) irradiation suffers from limited tissue penetration and may induce cellular apoptosis owing to its relatively high photon energy. In contrast, near‐infrared (NIR) light is safer and possesses superior tissue penetration. Rare‐earth UCNPs are luminescent materials capable of converting absorbed low‐energy infrared photons into high‐energy ultraviolet/visible photons [21]. Especially, lanthanide‐doped UCNPs (NaYF_4_:20% Yb, 0.5% Tm) could convert 980 nm NIR light into 365 nm UV light [22]. Accordingly, UCNPs (NaYF_4_:20%Yb,0.5%Tm) were selected in this study to enable NIR‐triggered stimulation of exosome secretion. The morphology of the UCNPs was first characterized by TEM. The results revealed that UCNPs had a particle size of approximately 20–30 nm and a hexagonal crystalline structure (Figure 2A,B). UCNPs exhibited stability in aqueous solution, with the hydrodynamic diameter showing negligible changes within 7 days (Figure S1). Time‐resolved fluorescence spectroscopy showed that under 980 nm excitation, the UCNPs displayed an emission spectrum with a strong peak at approximately 365 nm, and the emission intensity increased in a concentration‐dependent manner. Subsequently, the optimal conditions for co‐culturing MSCs with UCNPs were investigated. Cell viability assessment using the CCK‐8 assay revealed that 50 µg/mL represented the maximum concentration of UCNPs that did not compromise MSC viability, and this concentration was therefore selected for subsequent experiments (Figure 2C). Then MSCs were co‐incubated with UCNPs at various safe concentrations for 24 h, after which the cells were collected by centrifugation and resuspended. Under 980 nm excitation, the emission intensity of MSC‐UCNPs increased progressively with increasing UCNP concentration, indicating a concentration‐dependent uptake of UCNPs by MSCs (Figure 2D). To evaluate the impact of NIR irradiation on exosome release, the cytotoxicity of varying laser power densities on mesenchymal stem cells (MSCs) was first evaluated. The results showed that laser power density less than or equal to 1 W exerted no detectable cytotoxic effects on MSCs, whereas power density exceeding 1 W resulted in a significant decline in cellular viability (Figure 2E). Subsequently, the correlation between laser power density and exosome release from MSCs was explored. MSC‐UCNPs were subjected to 980 nm laser irradiation at graded power densities (0, 0.5, and 1 W) for a fixed duration of 30 min, with results indicating that exosome secretion reached the maximum level at the power density of 1 W (Figure 2F). Then, MSC‐UCNPs were irradiated with 980 nm light for varying durations, and cell supernatants were collected to isolate the released exosomes. Exosome production peaked at 30 min of irradiation, showing a 2.7‐fold increase compared to the control group. No significant differences were observed in the properties of the generated exosomes (Figure 2G–I). TEM was employed to directly visualize the endocytosis of UCNPs by MSCs and the dynamic process of exosome release. Compared with the UCNP‐free group, in the MSC‐UCNPs group, UCNPs were encapsulated within intracellular membrane‐bound structures, which may originate from lysosomes. Following 980 nm laser irradiation, numerous multivesicular bodies (MVBs) were distributed intracellularly without extracellular release, whereas a certain number of exosomes were scattered outside the cell membrane (Figure 2J). These observations validated that 980 nm laser irradiation can stimulate UCNPs to emit 365 nm ultraviolet light, thereby promoting exosome release from MSCs.

The release of extracellular vesicles (EVs) could be regulated by multiple factors. For instance, chronic inflammatory diseases, ultraviolet radiation [20], and pulsed ultrasound [23] can all promote EV release by inducing oxidative stress pathways. Previous studies have demonstrated that 980 nm NIR laser irradiation can promote exosome release from MSC‐UCNPs. Building on these findings, we aimed to further explore the molecular pathways underlying this exosome release process. Under optimized irradiation parameters, the generation and accumulation of ROS in MSC‐UCNPs were detected at 1, 4, 8, and 24 h post‐irradiation. The positive control confirmed the sufficient activation of ROS (Figure S2A). As shown in Figure 2K,L, NIR irradiation induced the activation of the oxidative stress pathway in MSC‐UCNPs. Compared with the MSC‐UCNPs without laser irradiation and the blank control group, the MSC‐UCNPs + Laser group exhibited significantly elevated ROS levels at all detected time points. The ROS level in the irradiated group reached the maximum at 1 h after 980 nm laser exposure, followed by a gradual decrease over time. This phenomenon may be attributed to the expression of superoxide dismutase 1 (SOD1) and SOD3 in MSCs. Furthermore, MSC‐EVs enhance SOD1 and SOD3 levels, thereby further promoting the restoration of ROS levels [24]. The magnitude of ROS elevation remained within a physiologically tolerable range, consistent with the absence of detectable cytotoxicity, indicating that the induced oxidative stress was mild and well tolerated by MSCs. Furthermore, to investigate the impact of oxidative stress on MSCs, comet assays and senescence‐associated β‐galactosidase (SA‐β‐gal) staining were performed. The results demonstrated that no significant DNA damage or cellular senescence was observed in the MSC‐UCNPs + laser irradiation group (Figure 2M–P), suggesting the biosafety of UCNPs and laser irradiation. Notably, ROS have been shown to promote both the expression and stability of hexosaminidase B (HEXB) within cells. HEXB could regulate lysosome‐associated membrane protein 1 (LAMP1) to impair the fusion of multivesicular bodies (MVBs) with lysosomes, thereby increasing the release of EVs [25]. Lysosomes are pivotal organelles that participate in the regulation of exosome secretion. Importantly, the release of exosomes into the extracellular milieu is negatively correlated with the degree of co‐localization between MVBs and lysosomes within cells [26]. To directly visualize the regulatory effect of HEXB on LAMP1, cellular immunofluorescence assays were performed to evaluate the co‐localization of multivesicular bodies (MVBs) and lysosomes (Figure 2Q). Compared to the untreated control group and the non‐irradiated MSC‐UCNPs group, the MSC‐UCNPs+Laser group exhibited diminished co‐localization between MVBs and lysosomes. In order to further determine the changes in HEXB and LAMP1, Western Blot assays were conducted. As shown in Figure 2R, compared to the non‐irradiated group, HEXB expression was significantly upregulated in MSCs, while LAMP1 expression was markedly downregulated. Except for the representative result figure, the other two groups of blot results and raw data were provided in Figure S2B,C. Collectively, the activation of MSC‐UCNPs via irradiation triggered intracellular oxidative stress. The increased ROS subsequently upregulated the expression and enhanced the stability of HEXB in MSCs, which in turn downregulated LAMP1 expression and compromised lysosomal function. As a result, the fusion between MVBs and lysosomes was attenuated, leading to reduced lysosomal degradation of MVBs and, ultimately, augmented exosome secretion from MSCs.

### 2.3 MSC‐Exo Enhance Self‐renewal and Osteogenic Commitment of MSCs

**FIGURE 3:**
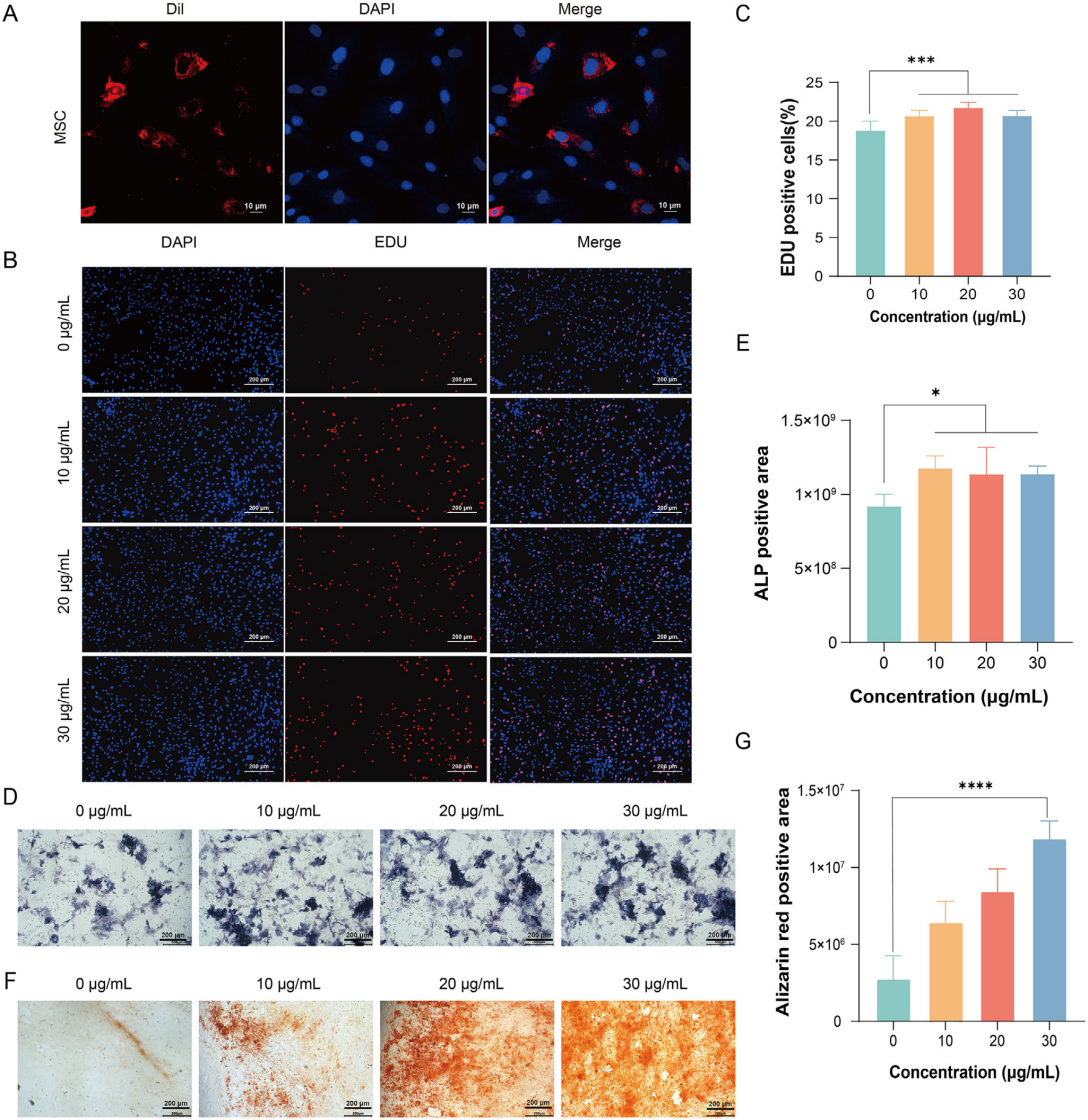
MSC‐Exo enhance self‐renewal and osteogenic commitment of MSCs. (A) Uptake of exosomes by MSCs (Scale bar: 10 µm). (B) Representative images of EDU assay of MSCs treated with different concentrations of MSC‐Exo (Scale bar: 200 µm). (C) Quantification analysis of percentage of EdU‐positive cells (*n* = 5), ***p < 0.001. (D) Representative images of ALP staining for MSCs treated with different concentrations of MSC‐Exo (Scale bar: 200 µm). (E) Quantitative analysis of ALP positive area calculated with ImageJ software (*n* = 5), *p < 0.05. (F) Representative images of alizarin red staining of MSCs treated with different concentration of MSC‐Exo (Scale bar: 200 µm). (G) Quantitative analysis of Alizarin red positive area (*n* = 5), ****p < 0.0001.

In bone defect repair, the differentiation of MSCs into osteoblasts is critical for effective bone regeneration [27]. Multiple factors have been reported to promote the osteogenic differentiation of MSCs, among which exosomes have attracted particular attention. These nanosized vesicles carry a diverse repertoire of bioactive molecules, including DNA, RNA, mRNA, lipids, metabolites, and proteins, and play pivotal roles in tissue regeneration and repair processes [28, 29]. To study the roles of MSC‐Exo on parental MSCs, the internalization of exosomes by MSCs was first investigated. As Figure 3A shows, confocal laser scanning microscopy (CLSM) images revealed clear co‐localization of DiI‐labeled exosomes with MSCs, confirming the efficient uptake of exosomes and providing a basis for subsequent functional studies. Then EdU staining was conducted to evaluate the effect of MSC‐Exo on MSC proliferation. The results demonstrated that MSC‐Exo at different concentrations could promote MSC proliferation to varying degrees (Figure 3B,C). Additionally, ALP and alizarin red staining were employed to assess the osteogenic differentiation of MSCs. Positive ALP staining indicates early‐stage osteogenic differentiation, with results showing that MSC‐Exo at different concentrations significantly enhanced ALP activity in MSCs (Figure 3D,E). Alizarin red staining, which detects calcium deposits, was used to evaluate the osteogenic mineralization of MSCs. MSC‐Exo at various concentrations promoted the formation of mineralized nodules in MSCs, with nodule numbers increasing in a concentration‐dependent manner and being significantly higher than those in the control group (Figure 3F,G). These findings indicated that MSC‐Exo effectively promoted MSC proliferation and osteogenic differentiation.

### 2.4 MSC‐Exo Promoted Osteoblast Maturation via Wnt/β‐Catenin Pathway

**FIGURE 4:**
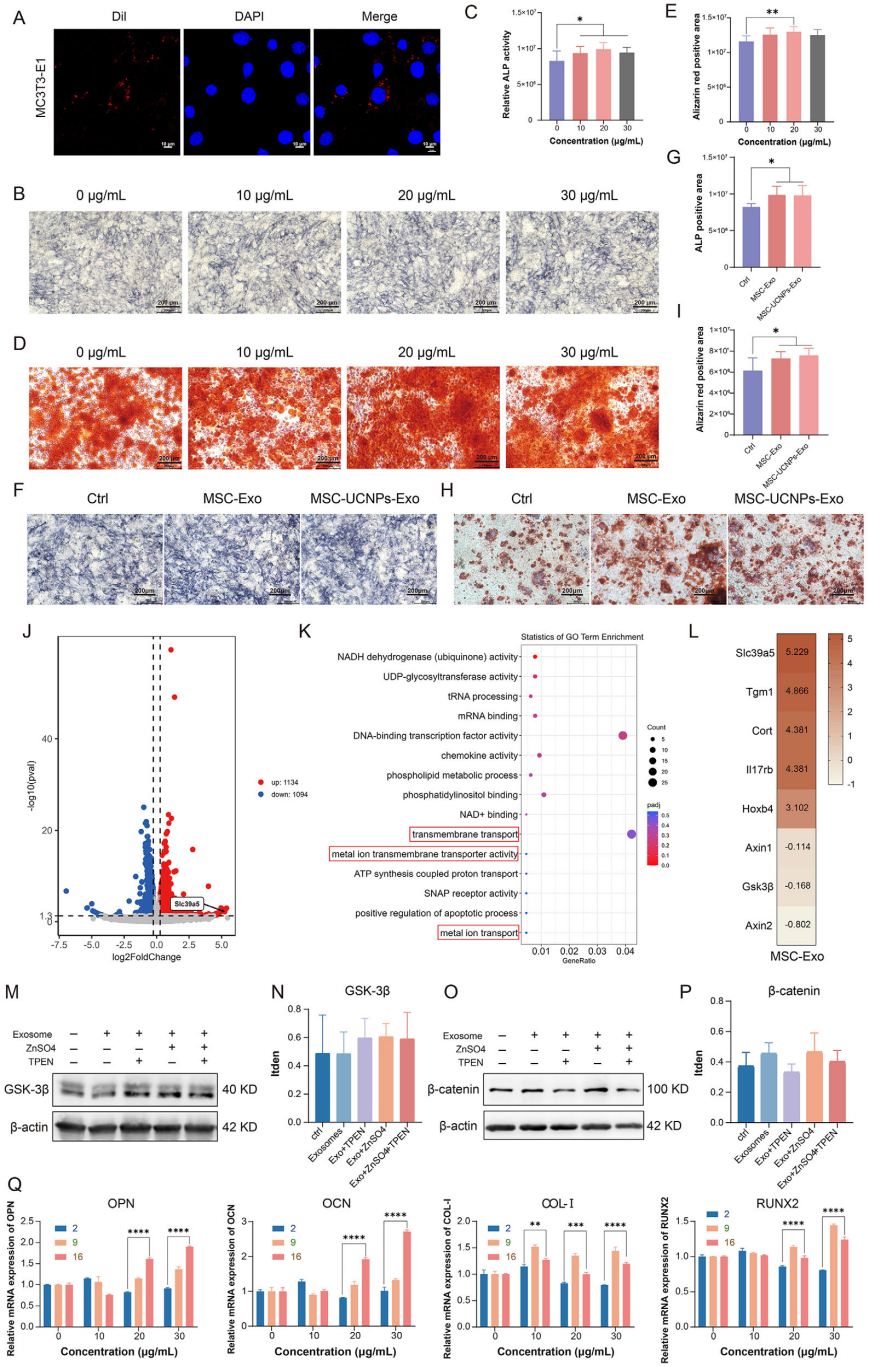
MSC‐Exo promoted osteogenic differentiation and maturation of MC3T3‐E1 cells. (A) Uptake of exosomes by MC3T3‐E1 cells (Scale bar: 10 µm). (B) Representative images of ALP staining of MC3T3‐E1 cells treated with different concentrations of MSC‐Exo (Scale bar: 200 µm). (C) Quantitative analysis of ALP‐positive area calculated with ImageJ software (*n* = 5), *p < 0.05. (D) Representative images of alizarin red staining of MC3T3‐E1 cells treated with different concentrations of MSC‐Exo (Scale bar: 200 µm). (E) Quantitative analysis of Alizarin red positive area calculated with ImageJ software (*n* = 5), **p < 0.01. (F) ALP staining of MC3T3‐E1 cells treated with exosomes from different sources (Scale bar: 200 µm). (G) Quantification of positively stained areas in ALP staining (*n* = 5), *p < 0.05. (H) Alizarin red staining of MC3T3‐E1 cells treated with exosomes from different sources (Scale bar: 200 µm). (I) Quantification of positively stained areas in alizarin red staining (*n* = 5), *p < 0.05. (J) Volcano plots showing the DEGs between control and MSC‐Exo groups. Blue indicates downregulated genes; Red indicates upregulated genes. (K) GO enrichment analyses of the DEGs in RNA‐seq data showing the key upregulated biological process in the MSC‐Exo group. (L) DEGs related to tissue repair of MSC‐ Exo group. (M) Representative images of a Western blot showing the expression levels of GSK‐3β, normalized to the expression of β‐actin. MC3T3‐E1 cells were cultured with MSC‐ Exo (50 µg/mL), ZnSO4 (10 µM), and TPEN (1 µM) for 48 h. (N) Quantitative analysis of GSK‐3β and β‐actin expression (*n* = 3). (O) Representative images of western blot showing the expression levels of β‐actin and normalized to the expression of β‐actin. (P) Quantitative analysis of β‐catenin expression (*n* = 3). (Q) RT‐qPCR analysis of the relative expression of osteogenic‐related genes (OPN, OCN, COL‐I, and RUNX2) in MSC‐Exo‐treated MC3T3‐E1 cells (*n* = 3), **p < 0.01, ***p < 0.001, ****p < 0.0001.

We then investigated the regulatory role of MSC‐Exo in promoting osteogenic differentiation and maturation at the osteoblast level. Firstly, confocal imaging confirmed that exosomes could be efficiently internalized by MC3T3‐E1 osteoblasts (Figure 4A). To systematically evaluate the osteogenic effect of MSC‐Exo on MC3T3‐E1 cells, ALP staining revealed that exosomes at different concentrations enhanced the positive staining intensity of osteogenic differentiation in MC3T3‐E1 cells, indicating relatively active osteogenic differentiation activity (Figure 4B,C). For assessing late‐stage osteogenic maturation, alizarin red staining, which is used to detect calcium deposition‐ demonstrated that MSC‐Exo promoted osteogenic mineralization of MC3T3‐E1 cells, as calcium deposits were stained dark red, confirming that the cells had achieved osteogenic maturation (Figure 4D,E). Furthermore, both MSC‐derived exosomes and exosomes obtained from infrared‐irradiated MSC‐UCNPs were compared. The exosomal marker analysis indicated that the two exosomes expressed CD9, CD63, and TSG101 (Figure S3). Meanwhile, both exosomes enhanced osteogenic differentiation and maturation of MC3T3‐E1 cells, with no statistically significant differences observed between the two groups (Figure 4F–I).

To further elucidate the molecular mechanisms underlying this process, RNA sequencing (RNA‐seq) analysis was performed on MC3T3‐E1 cells after 7 days of treatment. Differentially expressed genes (DEGs) between the control group and the MSC‐Exo‐treated group were analyzed. Volcano plot analysis revealed that, relative to the control group, 1134 genes were upregulated and 1094 genes were downregulated in MC3T3‐E1 cells treated with MSC‐Exo (Figure 4J). Notably, SLC39A5, a gene specifically associated with zinc ion transport, exhibited significant upregulation in the MSC‐Exo group. Consistently, Gene Ontology (GO) enrichment analysis demonstrated a significant enrichment of DEGs associated with the biological process of metal ion transport (Figure 4K). SLC39A5 functions as a zinc ion transporter, and its expression level modulates intracellular zinc concentrations, thereby non‐competitively inhibiting GSK‐3β and activating the Wnt/β‐catenin pathway [30]. Additionally, several tissue repair‐related genes, including Tgm1, Cort, Il17rb, and Hoxb4, were also upregulated (Figure 4L). Tgm1 encodes transglutaminase, which enhances bone mechanical strength via enzymatic cross‐linking and promotes osteogenic differentiation of osteoblasts [31]. Cort encodes cortactin, a cytoskeletal protein that has been reported to promote osteogenic differentiation via activation of the mTOR signaling pathway [32]. Il17rb, a subunit of the IL‐17 receptor, is involved in IL‐17‐mediated signal transduction, and IL‐17 maintains bone homeostasis by stimulating osteoblast differentiation and osteoclast formation [33]. Hoxb4 has been reported to enhance the reparative capacity of MSCs in lipopolysaccharide‐induced lung vascular endothelial cell injury [34]. Axin and GSK‐3β are core components of the APC/Axin/GSK‐3β/CK1 complex, which specifically sequesters intracellular β‐catenin and mediates its ubiquitination and degradation via the ubiquitin‐proteasome pathway, thereby negatively regulating the Wnt/β‐catenin pathway [30]. RNA‐seq analysis showed that the mRNA levels of Axin1, Axin2, and GSK‐3β were downregulated in the MSC‐Exo‐treated group (Figure 4L). Previous studies have demonstrated that activation of the Wnt/β‐catenin pathway in osteoblasts promotes osteogenic differentiation and maturation [35, 36]. In the present study, we further identified SLC39A5 as a positive regulator of the Wnt/β‐catenin pathway. As shown in Figure 4M–P, upregulation of SLC39A5 enhanced intracellular Zn^2+^ transport, resulting in significant downregulation of GSK‐3β expression and upregulation of β‐catenin expression. Conversely, treatment with the zinc chelator TPEN restored GSK‐3β expression and reduced β‐catenin levels, indicating that SLC39A5‐mediated Zn^2+^ transport inhibited GSK‐3β and activated the Wnt/β‐catenin pathway, thereby promoting osteogenic differentiation and maturation of MC3T3‐E1 cells. Western blot results for the three experimental groups are provided in the Supplementary Materials (Figure S4A,B). Finally, to further validate osteoblast maturation, RT‐qPCR was performed to assess the expression levels of mature osteoblast‐associated genes, including OPN, OCN, COL‐I, and RUNX2. The results showed that MSC‐Exo treatment at concentrations of 20 and 30 µg/mL significantly upregulated the expression of these osteogenic markers (Figure 4Q).

### 2.5 Preparation and Characterization of Sodium Alginate‐hyaluronic Acid‐Calcium Alginate Hydrogel

**FIGURE 5:**
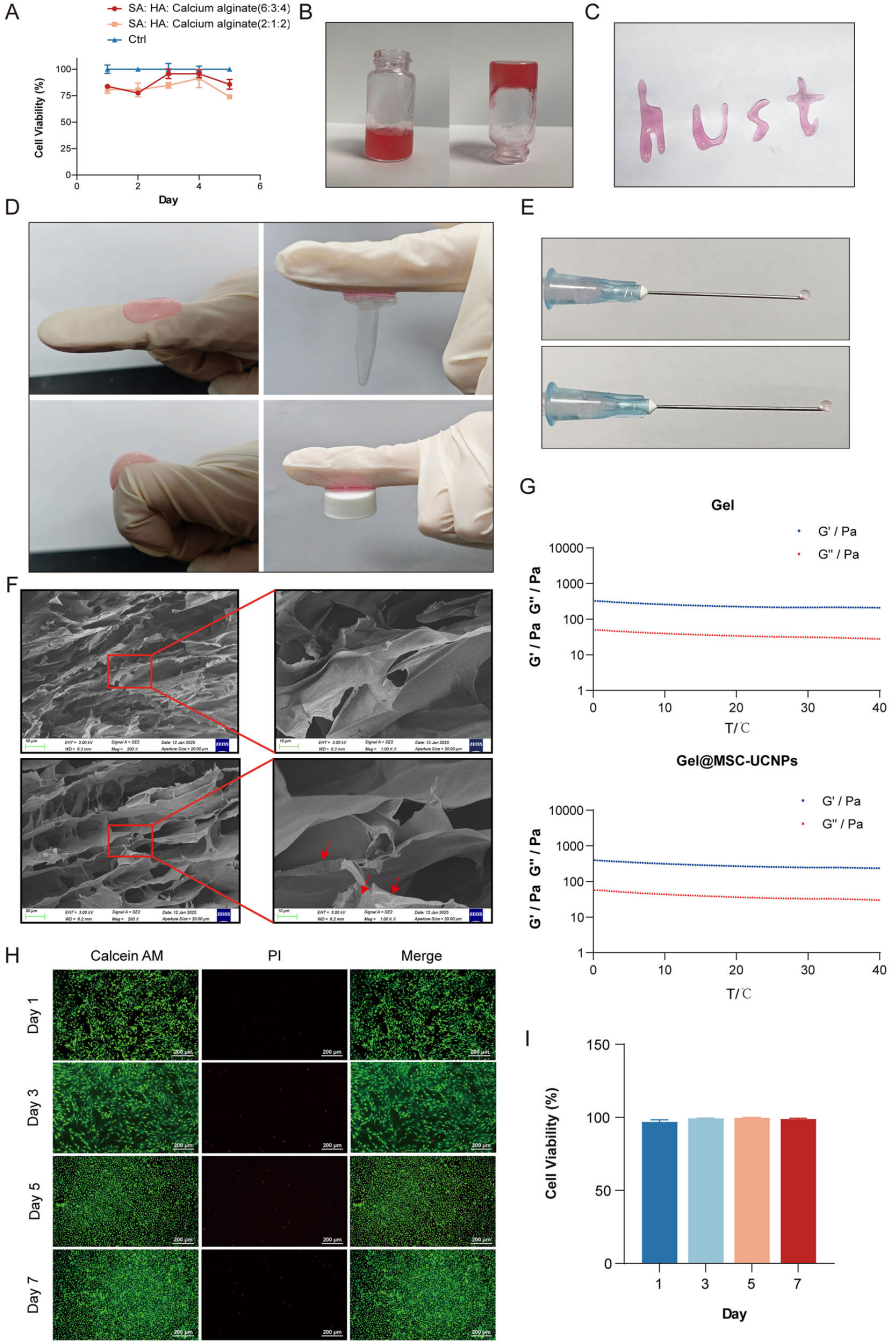
Preparation and characterization of MSC‐laden alginate‐hyaluronic acid‐carbonated alginate hydrogel. (A) Investigation on the cell compatibility of different ratios of SA‐HA‐calcium alginate composites (*n* = 3). (B) Formation of unloaded hydrogel. (C) Shape remodeling and adaptability of Gel. (D) Adhesion to the finger with different bending angles (left) and adhesive capacity on different substrates (right). (E) Injectability of Gel through the syringe. (F) SEM images of Blank‐Gel and Gel@MSC‐UCNPs. The red area indicates the magnified region. The red arrows indicate the MSCs. Scale bars are shown on each image respectively. (G) The rheological behavior of blank hydrogel (up) and MSC‐UCNPs‐loaded hydrogel (down) was tested by rheometer. (H) Immunofluorescence images of live and dead cells stained with MSC in alginate hydrogel. Scale bar:200 µm. (I) Statistical analysis of MSCs viability was performed at 1, 3, 5, and 7 days post‐treatment in the Gel (*n* = 5).

To address the rapid in vivo clearance of directly transplanted cells, a hydrogel system was employed as a carrier for MSC‐UCNPs. Sodium alginate (SA) hydrogel, a natural polysaccharide, has been widely applied in biomedical research due to its excellent cell biocompatibility, biodegradability, and ability to promote tissue healing [37, 38]. Hyaluronic acid (HA) was incorporated to provide an appropriate loading microenvironment. As a key component of the extracellular matrix, hyaluronic acid can mimic the in vivo cellular niche [39]. Therefore, SA, calcium alginate, and HA were combined at different mass ratios to optimize the microenvironment for MSC viability. The cytotoxicity of different formulations toward MSCs was evaluated using CCK‐8 assays. The results showed that the formulation containing SA, HA, and calcium alginate at a mass ratio of 6:3:4 maintained approximately 90% cell viability (Figure 5A). Accordingly, this optimal ratio was selected for subsequent experiments. The physicochemical properties of the sodium alginate‐based hydrogel, including moldability, remoldability, adhesiveness, and injectability, were systematically characterized. After gelation, the hydrogel exhibited a non‐flowable state with a uniform texture and high transparency (Figure 5B). Notably, the hydrogel could be shaped to form the word “hust”, demonstrating its excellent shape adaptability and suitability for conformal coverage of irregular wound surfaces (Figure 5C). The hydrogel was also capable of adhering to fingers and deforming synchronously with finger movement, indicating good flexibility. Additionally, its strong adhesiveness was confirmed by stable attachment to various materials (Figure 5D). Both blank hydrogels and MSC‐loaded hydrogels could be successfully injected through a 2 mL syringe, highlighting their potential for minimally invasive local administration (Figure 5E). Scanning electron microscopy (SEM) images further revealed the porous structure of the hydrogel. In contrast to the smooth surface of the blank hydrogel, Gel@MSCs exhibited a rougher surface due to the attachment of cells to the gel matrix (Figure 5F). Rheological analysis was conducted to determine the storage modulus (G') and loss modulus (G'') of the hydrogel (Figure 5G). Both the blank hydrogel and Gel@MSCs were identified as viscoelastic semi‐solid materials with inherent viscosity, indicating their ability to immobilize MSCs and enable sustained release. Cell compatibility was further assessed using live/dead cell staining. Calcein AM was used to label viable cells with green fluorescence, while propidium iodide (PI) stained dead cells with red fluorescence. MSC viability was reflected by analyzing the ratio of Calcein AM‐positive to PI‐positive cells. Viability assessments were carried out at 1, 3, 5, and 7 days after cell seeding in the hydrogel. After 7 days, the live/dead ratio of MSCs remained above 90%, demonstrating that the sodium alginate‐based hydrogel exhibited excellent cytocompatibility and supported long‐term cell survival (Figure 5H,I).

### 2.6 The Exosome Release From MSC‐UCNPs In Situ

**FIGURE 6:**
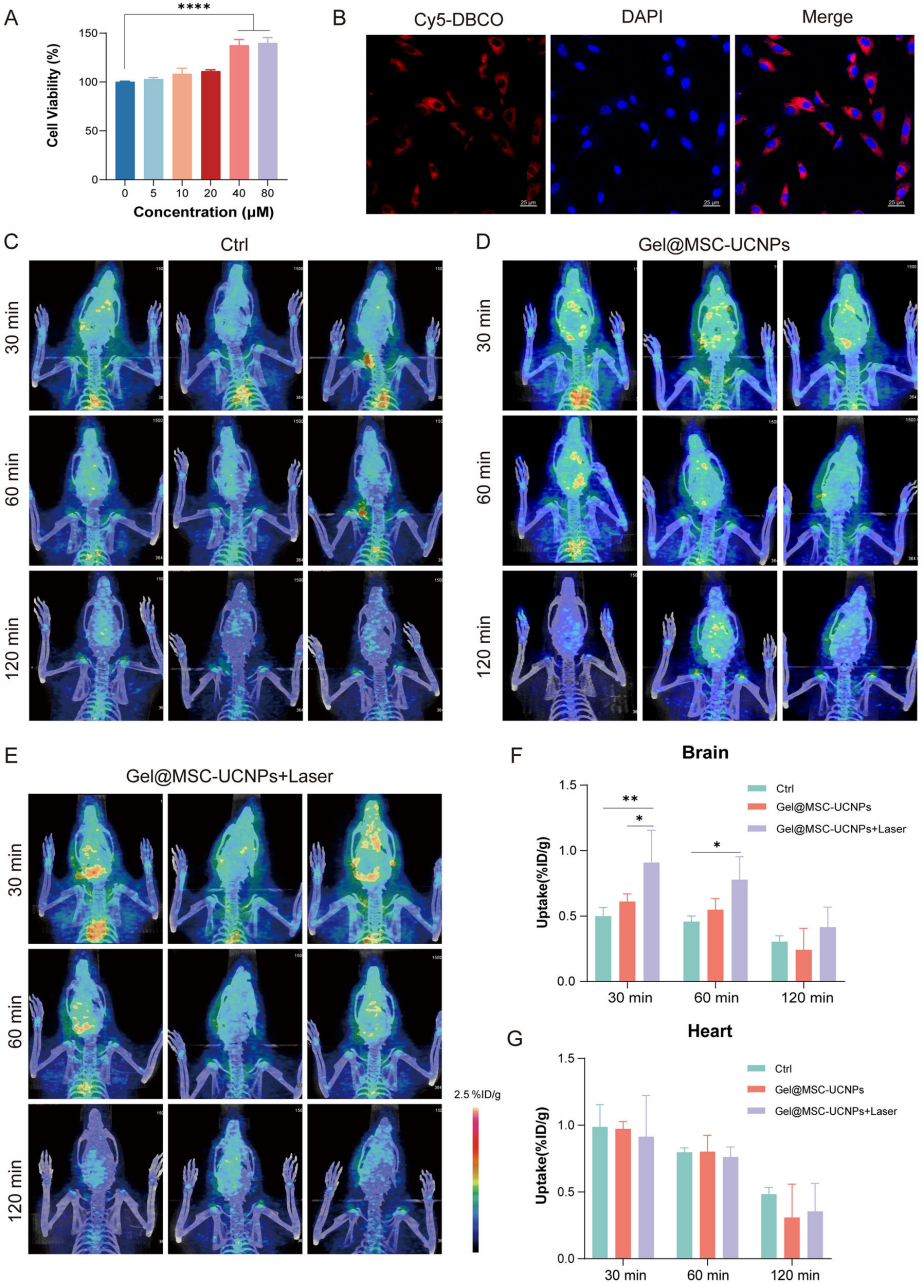
The exosome release from MSC‐UCNPs in situ. (A) Verification of the biocompatibility of Ac4ManNAz with MSCs (*n* = 3), ****p < 0.0001. (B) The covalent binding of CY5‐DBCO to nitroso‐labeled MSCs in vitro. (C–E) PET‐CT imaging enabled the detection of local exosome release in vivo. (F) Statistical analysis of ^68^Ga‐based brain tracking signals released by exosomes (*n* = 3), *p < 0.05, **p < 0.01. (G) Statistical analysis of ^68^Ga signals in the heart/blood pool (*n* = 3).

The aforementioned cellular experiments have confirmed that MSC‐UCNPs can secrete an increased quantity of exosomes under 980 nm irradiation. To further explore exosome release from MSC‐UCNPs in vivo, the released exosomes were tracked using Positron Emission Tomography–Computed Tomography (PET‐CT) imaging. Ac4ManNAz, a metabolic glycoprotein labeling reagent containing an azide group, can be internalized by cells, modify intracellular proteins, and be presented on the cell surface. The azide groups can then covalently conjugate with dibenzocyclooctyne (DBCO)‐modified probes via bioorthogonal click chemistry, enabling in vivo imaging and quantitative detection in animal models [40]. ^68^Ga‐DBCO was used as a tracer to track in situ exosome release. Firstly, the cytocompatibility of Ac4ManNAz was evaluated. As shown in Figure 6A, Ac4ManNAz exhibited no detectable cytotoxicity toward MSCs and even promoted cell proliferation. The binding between Ac4ManNAz and CY5‐DBCO was verified at the cellular level in vitro. CLSM revealed good co‐localization of CY5‐DBCO and Ac4ManNAz‐labeled MSCs (Figure 6B). Furthermore, exosomes secreted by these labeled MSCs were collected and analyzed by flow cytometry. The results showed that approximately 70% of the released exosome showed fluorescent signal, indicating the retention of Ac4ManNAz (Figure S5). Rats with cranial defects were then randomly divided into three groups: the control group, the Gel@MSC‐UCNPs group, and the Gel@MSC‐UCNPs + Laser group. Following 24 h of laser irradiation, each group of rats received a tail vein injection of ^68^Ga‐DBCO, and PET imaging was performed to detect local exosome release at 30, 60, and 120 min (Figure 6C–E). As the in vivo circulation time of ^68^Ga‐DBCO increased, ^68^Ga gradually decayed, leading to a progressive weakening of the signal. At 30 and 60 min, exosome signals in the defect region of the Gel@MSC‐UCNPs + Laser group were the strongest, significantly higher than those in the control group and the Gel@MSC‐UCNPs group. By 120 min, ^68^Ga had undergone two half‐lives, resulting in no significant differences in the detected signals across the groups (Figure 6F). Additionally, quantitative analysis of cardiac PET signals, which represent the blood pool activity, revealed no statistically significant differences among the groups, indicating comparable systemic tracer distribution and clearance (Figure 6G). As we injected equal amounts of cells in two MSC‐UCNPs groups, the signal of local accumulation was similar. Notably, the Ac4ManNAz‐retaining exosome could infuse outside the hydrogel to react with ^68^Ga‐DBCO in blood circulation. Herein, the enhanced PET signal could be attributed to the increased release of exosomes in situ.

### 2.7 The Reconstruction of Craniofacial Bone Defect by Light‐switched MSCs

**FIGURE 7:**
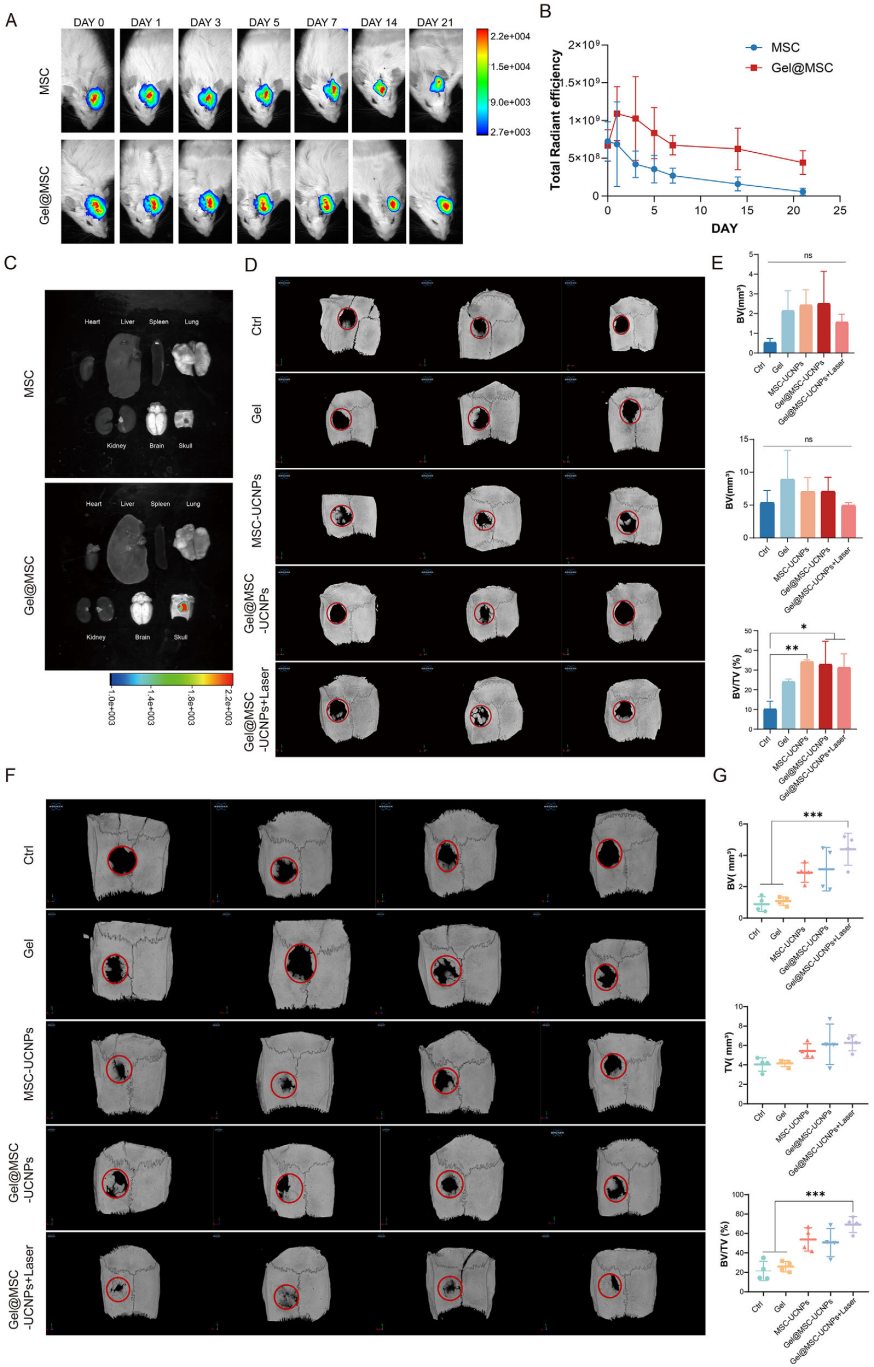
MSCs promoted the bone repair and regeneration in a rat model of cranial defect. (A) Retention of DiR‐labelled MSCs within 21 days. (B) Statistical analysis of DiR‐labelled MSCs that remain localized in the region of the rat skull (*n* = 3). (C) Retention of MSCs in vivo organs on day 21 (Up: free MSCs administration group; Down: Gel@MSCs administration group). (D) Micro‐CT scanning of rat calvarial defect repair at 4 weeks post‐administration. (E) Statistical analysis of BV, TV, and BV/TV data of 4 weeks (*n* = 3), *p < 0.05, **p < 0.01. (F) Reconstructed images of micro‐CT of skull at the eighth week. (G) The bone metabolism‐related indexes, TV, BV, and TV/BV of 8 weeks in each group were evaluated(*n* = 4), ***p < 0.001.

Free MSCs are rapidly cleared following in vivo administration, which limits their accumulation at target sites and consequently compromises therapeutic efficacy. In contrast, hydrogel‐based MSC delivery systems can effectively delay MSC clearance. To evaluate the local retention of MSCs and Gel@MSCs at the defect site post‐injection, rats with cranial defects were divided into two groups and locally injected with either free MSCs or Gel@MSCs. MSCs were labeled with DiR for in vivo fluorescence tracking. As shown in Figure 7A,B, with increasing retention time in vivo, the fluorescence signal of free MSCs decreased markedly faster than that of Gel‐encapsulated MSCs. In addition, the overall signal intensity of free MSCs was significantly weaker than that observed in the Gel@MSCs group, indicating more rapid clearance of unprotected cells. The prolonged retention of MSCs is beneficial for enhancing their tissue repair potential. On day 21, a sustained and detectable level of MSC signal was retained at the rat calvarial implantation site in the Gel@MSCs‐administered group, with no signals detected in other organs (Figure 7C).

To investigate the role of MSCs and their paracrine exosomes in tissue regeneration and repair, a critical craniofacial defect model was established. Rats were randomly assigned to five experimental groups: control, Gel, MSC‐UCNPs, Gel@MSC‐UCNPs, and Gel@MSC‐UCNPs + Laser. Isolated skulls were subjected to Micro‐CT examination at weeks 4 and 8 post‐treatment. At the fourth week post‐treatment, the MSC‐UCNPs, Gel@MSC‐UCNPs, and Gel@MSC‐UCNPs + Laser groups exhibited significantly enhanced bone regeneration compared with the control and Gel groups. (Figure 7D,E). By 8 weeks, the Gel@MSC‐UCNPs + Laser group demonstrated the most pronounced bone repair effect, with newly formed bone occupying approximately 70% of the defect area, which was significantly higher than that observed in the other groups (Figure 7F,G). Furthermore, the quantitative analysis of reconstruction parameter (BV/TV) was performed. Compared with the control group, MSC‐UCNPs group showed approximately 2.5‐fold higher of BV/TV value, highlighting the effective regenerative capacity of MSCs. In contrast, when comparing Gel@MSC‐UCNPs with Gel@MSC‐UCNPs + Laser, the additional therapeutic benefit in the laser‐treated group is primarily associated with enhanced exosome‐mediated paracrine effects. Specifically, the BV/TV value of Gel@MSC‐UCNPs + Laser was approximately 1.4‐fold higher than that of Gel@MSC‐UCNPs. The direct photographs of freshly isolated rat calvariae at 8 weeks post‐intervention are presented in Figure S6. A preliminary qualitative assessment of mechanical properties, performed by manual palpation of harvested calvarial specimens, revealed no discernible difference in hardness between the regenerated defect site and the contralateral intact bone. These findings imply that the regenerated bone could achieve functional competence. Collectively, MSC‐driven tissue repair provides a stronger baseline contribution than exosomes alone, while MSC‐based regeneration and exosome‐mediated paracrine effect synergistically promote calvarial bone regeneration.

### 2.8 Histological and Immunohistochemical Analysis of Cranial Tissue

**FIGURE 8:**
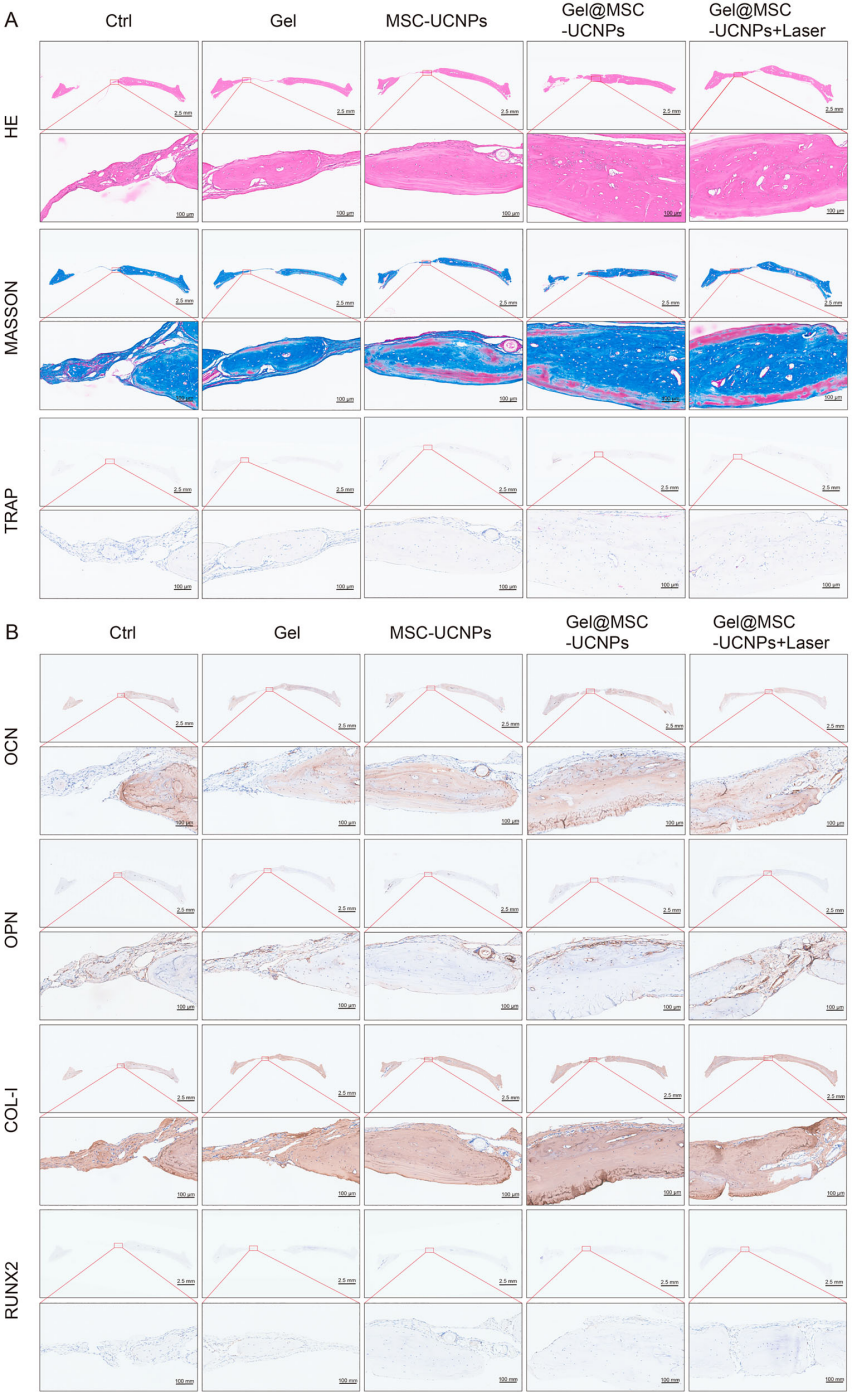
Histological and immunohistochemical analysis of cranial tissue. (A) Representative images of the HE staining, MASSON staining, and the TRAP staining. (B) Immunohistochemical staining of protein expression related to bone maturation.

Histological and immunohistochemical analyses of cranial tissues were further performed to evaluate bone regeneration and remodeling. Hematoxylin and eosin (H&E) staining and Masson's trichrome staining were used to assess new bone formation and collagen fiber deposition, respectively, while tartrate‐resistant acid phosphatase (TRAP) staining was applied to evaluate osteoclast activity. In the control group, no significant new bone formation was observed after 8 weeks. In contrast, all treatment groups exhibited varying degrees of bone regeneration. Among them, the Gel@MSC‐UCNPs + Laser group demonstrated the most pronounced formation of newly generated bone tissue and collagen fibers, indicating superior regenerative outcomes (Figure 8A). Immunohistochemical staining was also conducted on the extracted tissues from the isolated skulls to determine the expression of relevant osteogenic proteins in vivo. OCN, OPN, COL‐I, and RUNX2, which collectively serve as representative markers of osteogenic differentiation and bone maturation, were analyzed. Compared with the other groups, the Gel@MSC‐UCNPs + Laser group exhibited stronger and more widespread positive staining signals, suggesting enhanced osteogenic activity, accelerated bone maturation, and improved repair of cranial defects (Figure 8B). Moreover, the pathological damages of major tissues (brain, heart, liver, spleen, lung, and kidney) were examined by HE staining. The results showed that Gel@MSC‐UCNPs caused no apparent morphological alterations or tissue injury compared with the control group (Figure S7).

## 3 Discussion and Conclusion

The regeneration of critical‐sized craniofacial bone defects remains a formidable challenge in orthopedics and regenerative medicine, primarily due to the paucity of residual bone tissue and the limited intrinsic regenerative capacity of the defect microenvironment. Bone regeneration is a highly orchestrated, multistage biological process involving hematoma formation, inflammation, fibrous and cartilaginous callus development, ossification, and long‐term remodeling [41, 42]. Throughout these stages, MSCs function as central regulatory elements, responding to local biochemical and biophysical cues and differentiating into osteoblast lineage cells [43]. However, MSC‐derived osteoblasts initially exhibit an immature phenotype with limited matrix secretion and mineralization capacity. It is crucial to transform these nascent cells into mature cells that are highly active and capable of efficiently secreting and mineralizing the matrix [44]. Beyond their differentiation potential, MSCs exert profound therapeutic effects through paracrine signaling, particularly via the secretion of exosomes, which play a decisive role in promoting osteoblast maturation and functional integration [45]. Nevertheless, under physiological conditions, exosome secretion by MSCs is relatively inefficient. It has been reported that under low‐dose near‐ultraviolet light (∼365 nm) irradiation, paracrine secretion of exosomes by cells can be significantly increased [20]. Owing to its weak tissue penetration and safety concerns, ultraviolet light is restricted for in vivo application to a considerable extent. This limitation highlights the unmet need for a strategy that enables localized ultraviolet stimulation while preserving deep tissue penetration and biological safety. Upconversion nanoparticles (UCNPs) provide a compelling solution by converting deeply penetrating near‐infrared (NIR) light into higher‐energy visible or ultraviolet emissions. In particular, NaYF_4_: 20%Yb, 0.5%Tm represents a well‐established upconversion system capable of generating stable ∼365 nm emission via a multiphoton energy transfer process between Yb^3+^ sensitizers and Tm^3+^ activators [46, 47]. Therefore, UCNPs were intracellularly integrated into MSCs to construct light‐switchable stem cells, enabling spatially confined and intracellularly localized ultraviolet generation upon 980 nm irradiation. The near‐infrared light in the 700–1000 nm window exhibits minimal absorption by endogenous chromophores and reduced phototoxicity compared with shorter wavelengths, allowing efficient tissue penetration with negligible thermal damage under appropriate power densities.

Upon irradiation, the MSCs with UCNPs can be excited and emit 365 nm ultraviolet light. The ultraviolet light of 365 nm belongs to the UVA band, which possesses substantially lower photon energy than UVB and UVC. Unlike UVB, which directly induces DNA strand breaks and pyrimidine dimer formation, UVA primarily exerts its biological effects through indirect photochemical pathways [48]. Although UVA can stimulate endogenous photosensitizers such as riboflavin and porphyrins to generate ROS [49]; although the magnitude of ROS production under UVA exposure is markedly lower than that induced by UVB irradiation—reported to be approximately 40‐fold lower at equivalent doses [50]. Consistent with this, the UVA generated by intracellular UCNPs in our system induced a moderate and transient ROS elevation, sufficient to activate downstream signaling pathways without triggering oxidative stress‐mediated cytotoxicity. Mechanistically, the moderate ROS elevation stabilized β‐hexosaminidase subunit beta (HEXB), prolonging its intracellular half‐life and enhancing its functional activity [25]. As an important component of the key enzymes for decomposing lipids in the inner membrane of cells, the increased HEXB can lead to specific lipids, such as GM2 ganglioside, being unable to be degraded and accumulating in large quantities in lysosomes, thereby impairing lysosomal degradative capacity [51]. Lysosomes serve as key regulators of multivesicular body (MVB) turnover by mediating lysosome–MVB fusion [52]. Therefore, when the function of lysosomes is inhibited, their clearance effect on multivesicular bodies is decreased, and the ability of multivesicular bodies to generate exosomes is correspondingly increased. Notably, this regulatory mechanism operated within a controllable range as the lysosomal activity was modulated rather than completely suppressed, thereby avoiding catastrophic cellular dysfunction and preserving MSC viability.

Transcriptomic analysis further revealed that exosomes generated under light stimulation exerted distinct biological effects on recipient osteoblasts. Among the significantly upregulated genes, SLC39A5, a zinc transporter, played a pivotal role in osteoblast maturation. Elevated intracellular zinc levels noncompetitively inhibited glycogen synthase kinase‐3β (GSK‐3β), leading to suppression of the β‐catenin destruction complex composed of Axin, APC, and CK1α [53]. As a result, β‐catenin accumulated in the cytoplasm and translocated into the nucleus, where it activated TCF/LEF‐dependent transcription of osteogenic maturation markers such as OCN and COL‐I [54]. These findings underscore that MSC‐derived exosomes not only promoted osteogenic differentiation but also actively facilitated osteoblast functional maturation via Wnt/β‐catenin signaling pathway.

Due to the loss of bone tissue at the bone defect site, the direct administration of therapeutic agents is easily absorbed or cleared by the surrounding tissues. Therefore, an appropriate scaffold is needed to protect the drug while releasing it continuously. Hydrogels are composed of hydrophilic polymers. By adjusting the composition of the polymers and the crosslinking density, different mechanical properties (strength, elasticity, and toughness) can be customized, and they can also be matched with irregular defect areas to form any shape. In addition, hydrogels can be completely degraded after tissue repair, creating growth space for new tissues and eliminating the need for secondary surgical removal [55]. In this study, the alginate–hyaluronic acid hydrogel enabled prolonged retention of MSC‐UCNPs while allowing nutrient exchange, thereby facilitating on‐demand in situ exosome generation.

In conclusion, we developed a light‐switchable MSC platform through a simple and scalable strategy, enabling intracellular integration of UCNPs. Upon 980 nm near‐infrared irradiation, MSC‐UCNPs generated localized 365 nm UVA emission, which induced a mild and transient oxidative response without compromising cellular viability or osteogenic potential. This intracellularly confined photostimulation activated the ROS/HEXB/LAMP1 signaling cascade, partially suppressing lysosomal clearance of multivesicular bodies and thereby promoting sustained exosome biogenesis and secretion. The MSC‐derived exosomes not only enhanced osteogenic differentiation of parental MSCs but also promoted the maturation and functional activity of differentiated osteoblasts through zinc‐dependent inhibition of GSK‐3β and subsequent activation of the Wnt/β‐catenin signaling pathway. When embedded within an injectable hydrogel, MSC‐UCNPs exhibited good viability, prolonged retention in the defect site, and increased in situ exosome release. By synergistically integrating MSC‐based cell therapy with controllable exosome‐mediated paracrine signaling, this light‐responsive system significantly accelerated craniofacial bone defect regeneration. This work offers a new paradigm for regenerative medicine that bridges cellular therapy and cell‐free therapeutic modalities.

## 4 Experimental Section

### 4.1 Ethics Statement

Sprague–Dawley (SD) rats were purchased from Beijing Weitong Lihua Experimental Animal Technology Co., Ltd. (Beijing, China). The animal experiments were approved ([2024] IACUC Number: 4792) under the regulation of the Institutional Animal Care and Use Committee of Tongji Medical College, Huazhong University of Science and Technology.

### 4.2 Cell Culture

Bone marrow‐derived MSCs were isolated from the femora and tibiae of 3‐ to 4‐week‐old male SD rats. The rats were sacrificed and immersed in 75% ethanol for 5 min before bone tissue extraction. The femora and tibiae were dissected, and the bone marrow cavity was flushed with PBS using a disposable syringe. The flushed bone marrow was then ground through a 40‐µm strainer to obtain the cell suspension. After lysing the red blood cells, the resulting cells were cultured at the incubator at 37°C with 5% CO_2_. The MSCs from passages 3 to 5 were used for the subsequent experiments.

### 4.3 Isolation and Characterization of MSC‐Exo

MSC‐exosomes were isolated and purified by collecting the supernatant of MSCs (1 × 10^7^) using an exosome Isolation and Purification Kit (from Cell Culture Media) (UR52121, Umibio). The morphology of exosomes was observed under a transmission electron microscope (TEM, HITACHI, Japan). Exosome membrane proteins were quantified by the BCA Protein Assay kit. The particle size of exosomes and zeta potential were detected by Nano‐ZS dynamic light scattering (Malvern, UK).

### 4.4 The Release of MSC‐Exo Under 365 Nm UV Light

MSCs were seeded in 96‐well plates (irradiation height fixed at 20 cm) and exposed to 365 nm UV light for 0, 15, 30, 45, or 60 min, respectively, followed by 72 h of incubation. Exosomes were extracted from supernatants, with protein concentration, size distribution, and zeta potential determined. After determining the optimal irradiation time, the irradiation height was adjusted to 10 or 20 cm, respectively. The supernatant of MSCs was also extracted to isolate exosomes for the determination of protein concentration, particle size distribution, and zeta potential. The optimal UVA irradiation conditions for enhancing exosome production by MSCs were established.

### 4.5 Characterization of UCNPs

UCNPs (NaYF4: 20% Yb, 0.5% Tm) were purchased from Xi'an Ruixi Biological Technology Co., Ltd. A 2 mg/mL stock solution was added to 2–3 mL absolute ethanol, homogenized by ultrasonic dispersion, and a small aliquot was applied to a clean copper grid. After solvent evaporation, the morphology was observed by TEM. The UCNPs stock solution was diluted with PBS to 20 and 50 µg/mL, and the upconversion fluorescence spectra of these were measured using a time‐resolved fluorescence spectrometer (FluoroMax+, Horiba).

### 4.6 Preparation of MSC‐UCNPs

MSCs were co‐incubated with UCNPs, and the biocompatibility of UCNPs with MSCs was detected by CCK‐8; the optical density (OD) at 450 nm was measured by an enzyme‐labeled instrument (DR‐200Bs, India). Within the safe concentration range, MSCs were co‐cultured with UCNPs at various concentrations for 24 h. Uninternalized free UCNPs were removed by centrifugation, and the obtained MSC‐UCNPs were resuspended in PBS. The emission spectra of the cell suspension under 980 nm excitation were measured using atime‐resolved fluorescence spectrometer.

### 4.7 Light‐Switched Exosome Release From MSC‐UCNPs

At a fixed irradiation height of 20 cm, MSCs were exposed to 980 nm laser irradiation at power densities of 0, 0.5, and 1 W for a duration of 30 min. At the power densities of 1 W and a fixed height of 20 cm, MSCs were irradiated with a 980 nm laser for 0, 15, 30, and 45 min, respectively. After culturing for 72 h, the exosomes were extracted from the cell supernatant, and their protein concentration, particle size distribution, and zeta potential were measured.

### 4.8 Oxidative Stress Analysis of MSC‐UCNPs

MSCs were seeded in 24‐well plates and allocated into three groups: control, UCNPs‐treated, and UCNPs‐treated + Laser irradiation. The cells were incubated for 1, 4, 8, and 24 h, respectively. Subsequently, the supernatant was aspirated, and the cells were rinsed twice with PBS. A reactive oxygen species (ROS) fluorescent probe was then added, followed by incubation at 37°C in the dark for 30 min. Then the cells were harvested, and ROS activity was quantified by a flow cytometer (DxFLEX, BECKMAN COULTER, America). MSCs treated with hydrogen peroxide served as the positive control.

### 4.9 Immunofluorescence Staining

MSCs were seeded in confocal culture dishes and subjected to respective treatments: control, MSC‐UCNPs, and MSC‐UCNPs + Laser. The medium was aspirated, and the cells were rinsed twice with PBS. Cells were fixed with 4% PFA for 15 min, then 0.1% Triton X‐100 was added to induce permeabilization for 10 min at room temperature. Subsequently, the cells were blocked with 5% BSA at room temperature for 1 h. Then, cells were incubated with mouse CD63 monoclonal antibody (67605‐1‐Ig, Proteintech) and LAMP1 recombinant rabbit monoclonal antibody (HA722302, HUABIO) at 4°C overnight. Afterward, the cells were incubated with a mixture of goat anti‐mouse Alexa Fluor 488 secondary antibody (1:500 dilution) and goat anti‐rabbit Alexa Fluor 597 secondary antibody (1:500 dilution) for 1 h at room temperature. Lastly, the co‐localization of lysosomes and multivesicular bodies was observed by a confocal microscope.

### 4.10 The Proliferation of MSCs by EDU Assay

MSCs were seeded in a 96‐well plate at a density of 5000 cells/well. Four groups of MSC‐Exo with varying concentrations were co‐cultured with MSCs for 48 h. Subsequently, the medium was replaced with EDU‐containing medium, and cells were incubated for 2 h. EDU staining (R11053.9, RIBOBIO) was then performed following the manufacturer's protocol.

### 4.11 Alkaline Phosphatase (ALP) Staining and Alizarin Red Staining

The BCIP/NBT Alkaline Phosphatase Color Development Kit (C3206, Beyotime) and Alizarin Red Solution (ALIR‐10001, Cyagen) were used to evaluate the effects of MSC‐Exo on the osteogenic differentiation of MSCs and the maturation of osteoblasts. MSCs and MC3T3‐E1 cells were seeded in 24‐well plates and cultured for 24 h to allow adherence. Different concentrations of MSC‐Exo were added to each well, followed by 48 h of incubation. Subsequently, the medium was replaced with medium from the Rat Bone Marrow Mesenchymal Stem Cells Osteogenic Induction Differentiation Kit (RAXMX‐90021, OriCell) for further culture. ALP activity was assayed at 14 days, and calcified nodules were detected at 21 days. Staining results were observed under a microscope, and ImageJ was used for data analysis.

### 4.12 RNA‐Sequencing of MC3T3‐E1

MC3T3‐E1 cells were inoculated into 6‐well plates. Following cell adhesion, the cells were cultured for 7 days in two different types of media: normal culture medium and medium supplemented with 50 µg/mL MSC‐Exo. The medium was replaced every two days. Total RNA was extracted from MC3T3‐E1 cells and subjected to sequencing analysis on a specific platform. HISAT2 v2.1.0 was used to conduct differential analysis of gene expression. The criteria for selecting differentially expressed genes were: the fold change in expression |log_2_FoldChange| >1, and the significance padj ≤ 0.05. GO and KEGG pathway enrichment analyses were performed using clusterProfiler (v3.8.1), with *p*‐values calculated via hypergeometric distribution. Terms with adjusted *p*‐values < 0.05 were considered significantly enriched, enabling the identification of core biological functions of differentially expressed genes.

### 4.13 RT‐qPCR Assay of MC3T3‐E1

MC3T3‐E1 cells were seeded in 6‐well plates. After co‐incubation with MSC‐Exo for 48 h, the cells were collected for RT‐qPCR analysis (Archimed R4, RocGene, China). Total RNA was isolated from MC3T3‐E1 cells using Trizol (R0016, Beyotime). cDNA templates were synthesized in a 20 µL reaction mixture containing RNA and GeniuScript RT SuperMix with gDNA Eraser 2.0 (M5RT04, BIOEAST) using a thermal cycler. Subsequent RT‐qPCR was performed with 2× Universal HS SYBR qPCR Master Mix (M4QS07, BIOEAST). Glyceraldehyde‐3‐phosphate dehydrogenase (GAPDH) served as the endogenous reference gene for normalization of gene expression. All analyses were performed in triplicate. Primer sequences are included in Table S1.

### 4.14 Preparation and Characterization of Sodium Alginate‐hyaluronic Acid‐calcium Alginate Hydrogel

Sodium alginate (SA, S278630, Aladdin), hyaluronic acid (HA, H823435, Macklin), and calcium alginate (21054, Sigma‐Aldrich) were used for hydrogel preparation. Two hydrogel formulations with different proportions were prepared, and the CCK‐8 assay was used to assess their effects on MSC viability. SA, HA, and calcium alginate were dissolved in DMEM/F12 medium, then mixed to form a hydrogel with final concentrations of 1.5% (wt/vol), 0.75% (wt/vol), and 1% (wt/vol), respectively. MSCs were incorporated during the mixing of components to form MSC‐loaded hydrogel. The blank hydrogel and MSC‐loaded hydrogel were subjected to vacuum freeze‐drying, and their microstructure was characterized using a scanning electron microscope (ZEISS GeminiSEM 300, Germany). Additionally, the rheological properties of the hydrogels were assessed with a rheometer (TA DHR‐2, America). A frequency sweep test was performed to evaluate changes in storage modulus (G') and loss modulus (G''), using a shear frequency range of 0–100 Hz and a fixed strain of 1%.

### 4.15 Live/Dead Assay of MSCs in Hydrogel

A live/dead assay was performed to evaluate the cytocompatibility of the hydrogels. MSCs in the hydrogel were seeded in 24‐well plates. At 1, 3, 5, and 7 days, the cell viability was assessed using a calcein/PI staining kit (C2025S, Beyotime). Samples were observed and imaged under an inverted fluorescent microscope (IX73, OLYMPUS, Japan). ImageJ was used for data analysis.

### 4.16 The Monitoring of Exosome Release In Situ From MSC‐UCNPs

Ac4ManNAz (HY‐W728531, MedChemExpress) labeled MSCs released Ac4ManNAz‐labeled exosomes. Upon release from the hydrogels, these exosomes were immediately bound by DBCO (HY‐42973, MedChemExpress) conjugated radionuclide ^68^Ga, enabling their tracking. Three groups of rats with skull defects received treatments with PBS, Gel@MSC‐UCNPs, and Gel@MSC‐UCNPs followed by 980 nm laser irradiation, respectively. At 24 h post‐treatment, the rats were administered DBCO‐^68^Ga via tail vein injection. The released exosome signals were captured using the Trans PET Discoverist 180 (RAYCAN, China).

### 4.17 The Retention of MSC‐UCNPs In Vivo

To assess MSCs retention in vivo, MSCs were first labeled with DiR (HY‐D1048, MedChemExpress). DiR‐labeled MSCs were encapsulated in sodium alginate hydrogel and locally injected into the skull defect sites of rats. Meanwhile, a control group injected only with free MSCs was included for comparison with the Gel@MSCs group. Each animal received MSCs at a concentration of 10^6^ cells/mL. Imaging analysis was performed on both experimental groups at 0, 1, 3, 5, 7, 14, and 21 days using a Multimodal Live Animal Imaging System (Bruker, America).

### 4.18 Treatments for Skull Defect and Recovery Evaluation

The rats with skull defects were divided into five groups and subjected to corresponding treatments as follows: Control group, the Gel group, the MSC‐UCNPs group, the Gel@MSC‐UCNPs group, and the Gel@MSC‐UCNPs + Laser group. At weeks 4 and 8 post‐surgery, SD rats were euthanized. The isolated skulls were then harvested and fixed in 4% PFA. Micro‐CT (SkyScan 1176, Bruker, Germany) was employed to assess the repair progression of the skull defect area. The region of interest (ROI) was defined as a cylindrical volume (diameter: 5 mm, height: spanning the full thickness of the calvarial bone including the defect) centered on the craniotomy site. The cylinder axis was aligned perpendicular to the cranial surface. The ROI center was positioned using the original defect boundary identified by surgical drill marks as the reference point. To ensure unbiased evaluation, specimens were randomly coded by an independent researcher prior to scanning, and the Micro‐CT operator remained blinded to treatment groups throughout image acquisition and quantitative analysis. Parameters related to new bone formation at the defect sites were analyzed, including bone volume (BV), tissue volume (TV), and relative bone volume (BV/TV).

### 4.19 Histological and Immunohistochemistry Analysis

Rats were subjected to model induction and subsequently euthanized at eight weeks post‐administration. The skulls were dissected, fixed in 4% PFA, and then decalcified in EDTA‐based decalcification solution for 21 days. Following decalcification, the specimens were embedded in paraffin and processed for staining. Hematoxylin‐eosin (H&E) and Masson staining were utilized to assess skull regenerative and repair capacity, while tartrate‐resistant acid phosphatase (TRAP) staining was used to evaluate bone resorption and osteoclast activity. For immunohistochemistry, paraffin sections were incubated overnight at 4°C with primary antibodies against OCN (PB1008, Boster), OPN (83341‐1‐RR, PTG), RUNX2 (ab236639, Abcam), and Collagen I (ARG21965, Arigobio).

### 4.20 Statistical Analysis

Statistical analysis was performed using GraphPad Prism 8 software. A *p*‐value < 0.05 was considered statistically significant.

## Author Contributions

The manuscript was written through the contributions of all authors. All authors have given approval to the final version of the manuscript. C.S. performed conceptualization, supervision, review, and editing, validation. T.T.W. performed conceptualization, writing – original draft, supervision, review, and editing. Y.J.L. performed methodology, formal analysis, investigation, writing – original draft. S.M.W. performed review/editing. X.M.B. performed review/editing. L.Y.Z. performed review/editing. Y.W.L. performed review/editing. Z.W.F. performed review/editing.

## Conflicts of Interest

None of the authors have any potential conflict of interest to disclose.

## Supporting information




**Supporting File**: advs75519‐sup‐0001‐SuppMat.docx.

## Data Availability

The data that support the findings of this study are available from the corresponding author upon reasonable request.
