# Supplementary material for: Light‐Switched Mesenchymal Stem Cells for In Situ Exosome Amplification in Craniofacial Bone Defect Reconstruction
Source: Adv Sci (Weinh). 2026 May 6;13(42):e75519. doi: 10.1002/advs.75519 (PMC13335616; doi:10.1002/advs.75519)
Supplement: Supplementary file 1 — Supporting File: advs75519‐sup‐0001‐SuppMat.docx. [file ADVS-13-e75519-s001.docx]

**Supporting information**

**Light-switched mesenchymal stem cells for *in situ* exosome** **amplification in** **craniofacial bone defect** **reconstruction**

Tingting Wu^1,2,#^ , Yajing Liu^1,2,#^, Shuman Wang^1,2^, Xiaoming Bai^1,2^, Luyun Zhang^1,2^, Yuwei Liu^1,2^, Zhiwen Fu^1,2^, Chen Shi^1,2,3^*

^1^ Department of Pharmacy, Union Hospital, Tongji Medical College, Huazhong University of Science and Technology, Wuhan, Hubei, China.

^2^ Hubei Province Clinical Research Center for Precision Medicine for Critical Illness, Wuhan, Hubei, China.

^3^ Hubei Key Laboratory of Natural Active Polysaccharides, Union Hospital, Tongji Medical College, Huazhong University of Science and Technology, Wuhan, Hubei, China

*Corresponding author: Chen Shi ([whxhchen@163.com](mailto:whxhchen@163.com)).

^#^ These authors contributed equally to this work.

**1. Supporting methods**

**1.1 The observation of exosome release process from MSC-UCNPs**

Biological transmission electron microscopy (HT7800, Hitachi, Japan) was employed to visualize the exosome release process. MSC-UCNPs were irradiated with 980 nm laser at 20 cm distance for 30 min, followed by 48 h culture. Cell pellets were collected by centrifugation, fixed with 2.5% glutaraldehyde, and subjected to subsequent procedures including osmium tetroxide fixation, dehydration, infiltration, embedding, and sectioning. Finally, the cell samples were stained with uranyl acetate and lead citrate (uranium-lead double staining) prior to observation under the biological transmission electron microscope. Non-irradiated MSCs were set as the control group.

**1.2 Establishment of skull defect model**

Male SD rats aged 6-8 weeks old were used to establish the skull defect model. Briefly, rats were anesthetized *via* intraperitoneal injection of 2% sodium pentobarbital. The parietal skin was incised with scissors, and a 5-mm defect was created in the skull using a bine trephine. Finally, the skin incision was sutured with absorbable sutures.

**1.3 Western blotting assay**

MSCs and MC3T3-E1 cells were seeded into 6-well plates. MSCs were assigned to three groups: control, MSC-UCNPs, and MSC-UCNPs + Laser. Following 30 minutes of 980 nm laser irradiation, the MSC-UCNPs + Laser group and other groups were further cultured for 1 h. The cells were then harvested, and total protein content was quantified by BCA. Protein samples were denatured by boiling, which were subsequently used for Western blotting. The primary antibodies utilized in this experiment were HEXB rabbit polyclonal antibody (ER63676, HUABIO) and LAMP1 recombinant rabbit monoclonal antibody (HA722302, HUABIO). Band intensities of the target proteins were quantified using ImageJ software for data analysis.

MC3T3-E1 cells were divided into five groups: control, exosome (Exo)-treated, Exo + ZnSO₄-treated, Exo + TPEN-treated, and Exo + ZnSO₄ + TPEN-treated groups. The cells were subsequently harvested for Western blotting to assess the expression levels of glycogen synthase kinase-3β (GSK-3β) and β-catenin in the Wnt/β-catenin signaling pathway. The primary antibodies used were as follows: GSK-3β recombinant rabbit monoclonal antibody (SY28-03, HUABIO), β-catenin recombinant rabbit monoclonal antibody (SA30-04, HUABIO). The data analysis was performed using ImageJ software.

**2. Supporting table and figures**

Table 1: Primer sequence for qRT-PCR.

| Gene | Primer sequence | Base number |
| --- | --- | --- |
| OPN | F: AGCAAGAAACTCTTCCAAGCAA  R: GTGAGATTCGTCAGATTCATCCG | 22  23 |
| OCN | F: TCTGACCTCACAGATGCCAAG  R: AGGGTTAAGCTCACACTGCT | 21  20 |
| COL-Ⅰ | F: CCCTGGTCCCTCTGGAAATG  R: GGACCTTTGCCCCCTTCTTT | 20  20 |
| RUNX2 | F: AACGATCTGAGATTTGTGGGC  R: CCTGCGTGGGATTTCTTGGTT | 21  21 |
| GAPDH | F: TGTGTCCGTCGTGGATCTGA  R: TTGCTGTTGAAGTCGCAGGAG | 20  21 |


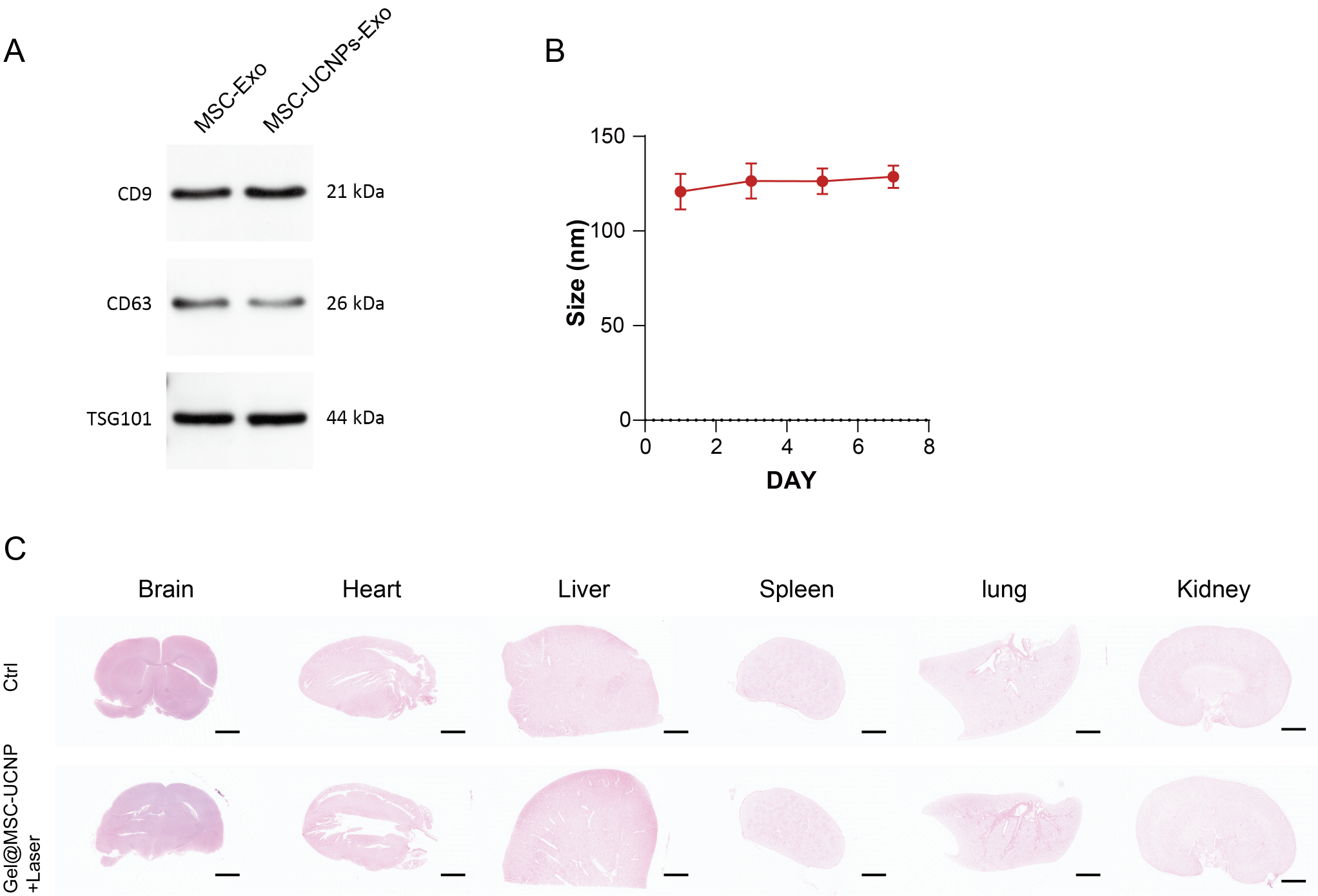


**Figure S1.** The change of hydrodynamic diameter of UCNPs measured by DLS.


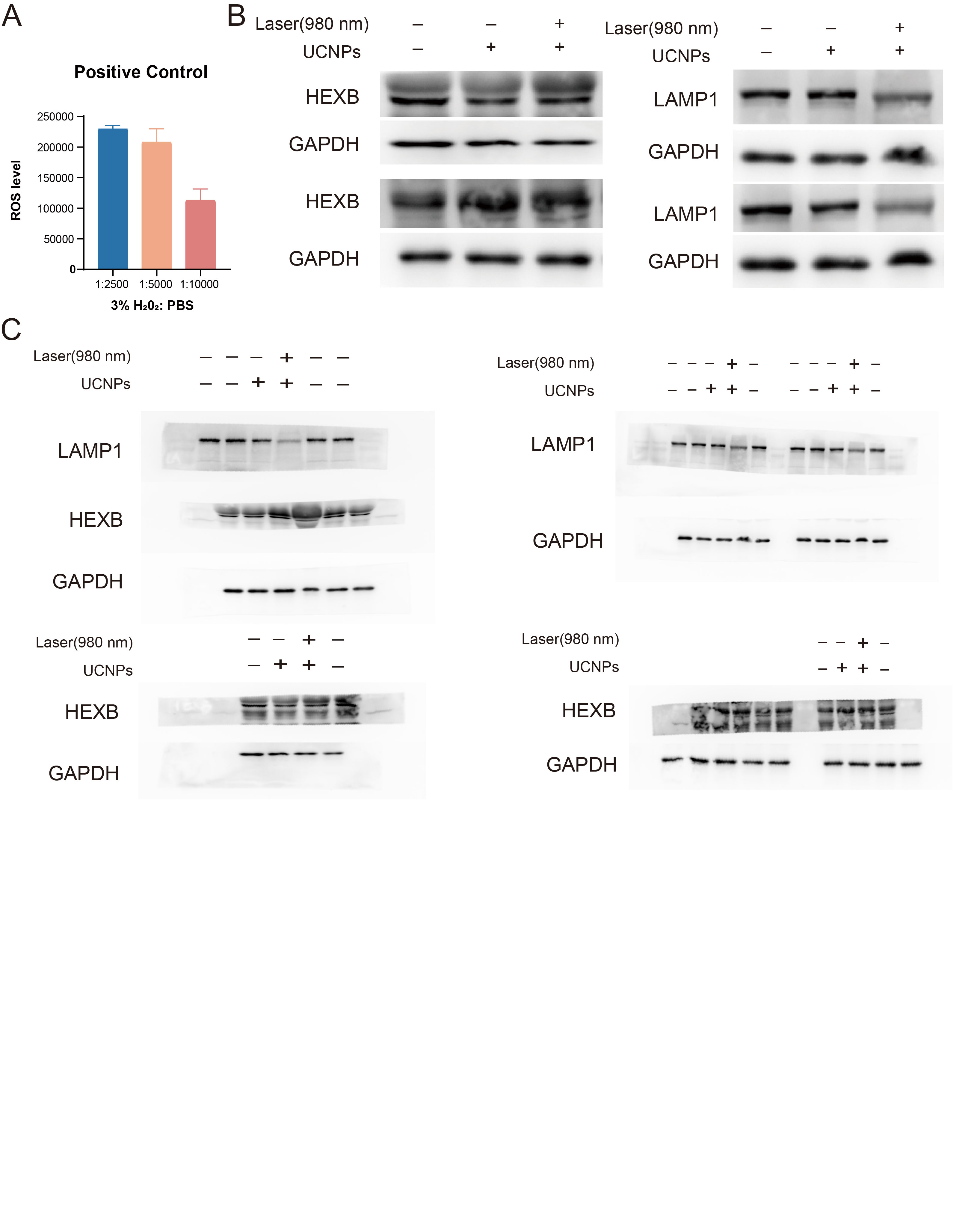


**Figure S2**. A) H₂O₂ positive control for oxidative stress (n=3); B) Images of HEXB and LAMP1 expression from another two groups of replicate experiments by western blotting; C) Original Western Blotting Images of HEXB and LAMP1.


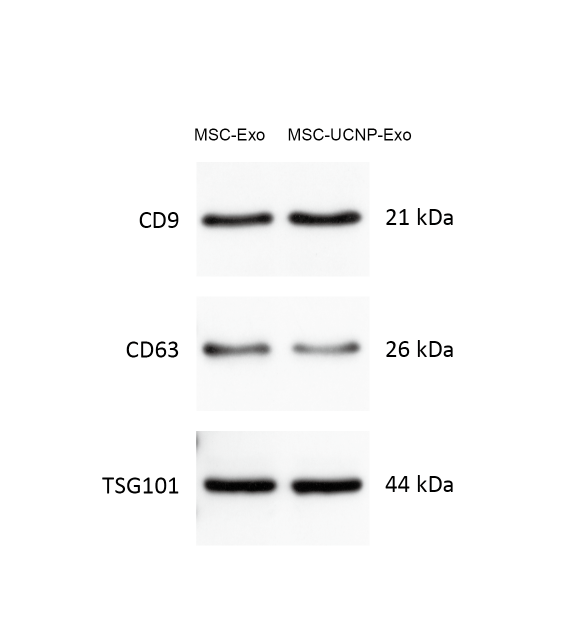


**Figure S3.** Identification of exosomal markers by western blot.


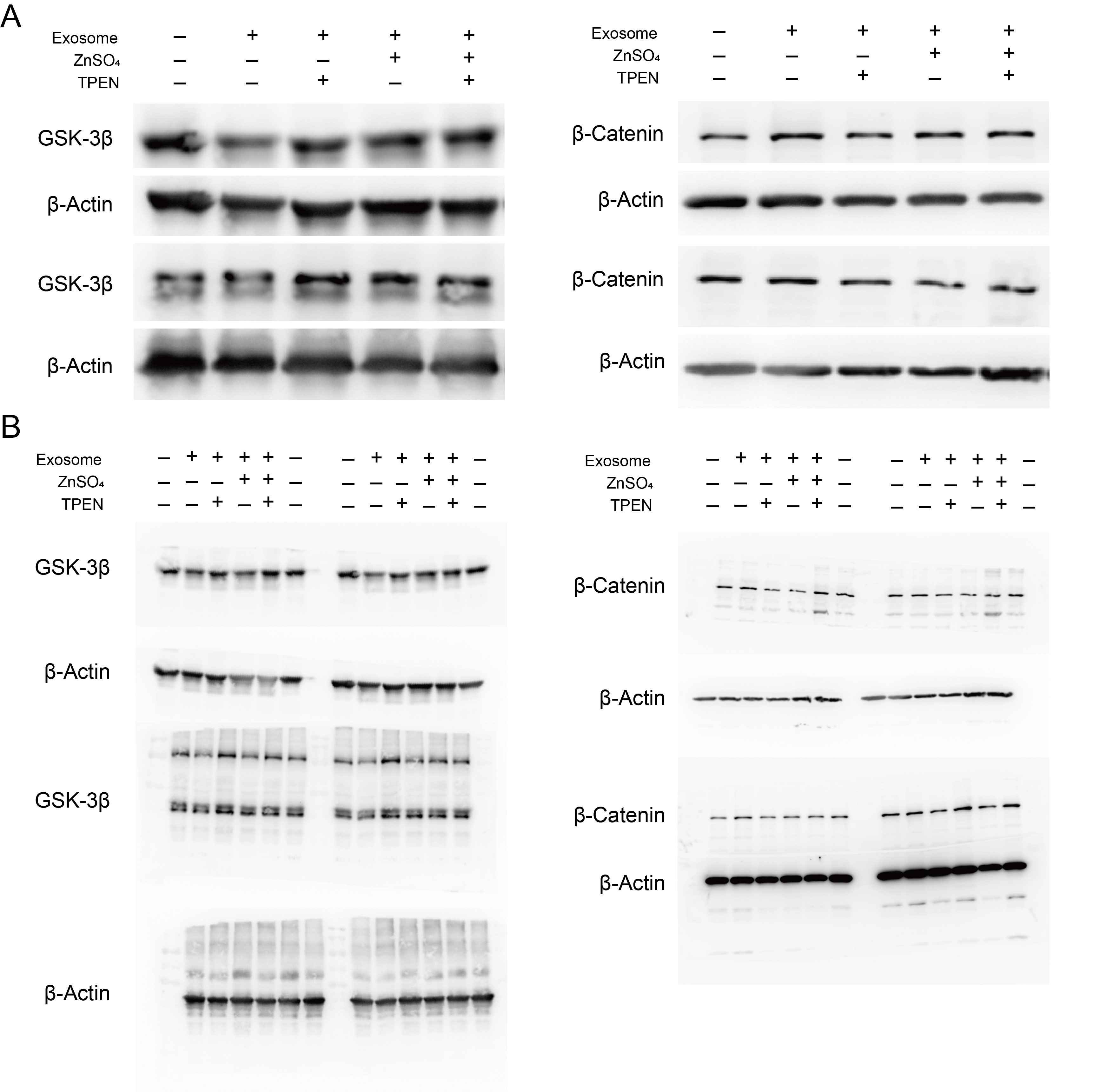


**Figure S4.** A) Additional Images of GSK-3β and β-Catenin Expression Levels (Normalized to β-Actin) from the Other Two Replicate Experiments by Western Blotting. B) Original Western Blotting Images of GSK-3β and β-catenin.


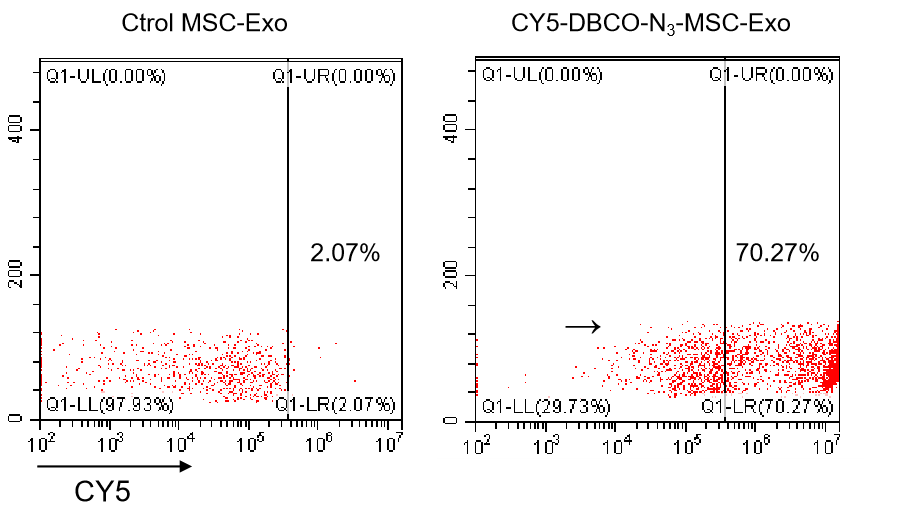


**Figure S5.** Flow cytometry analysis of CY5-DBCO-N_3_-MSC-Exo.


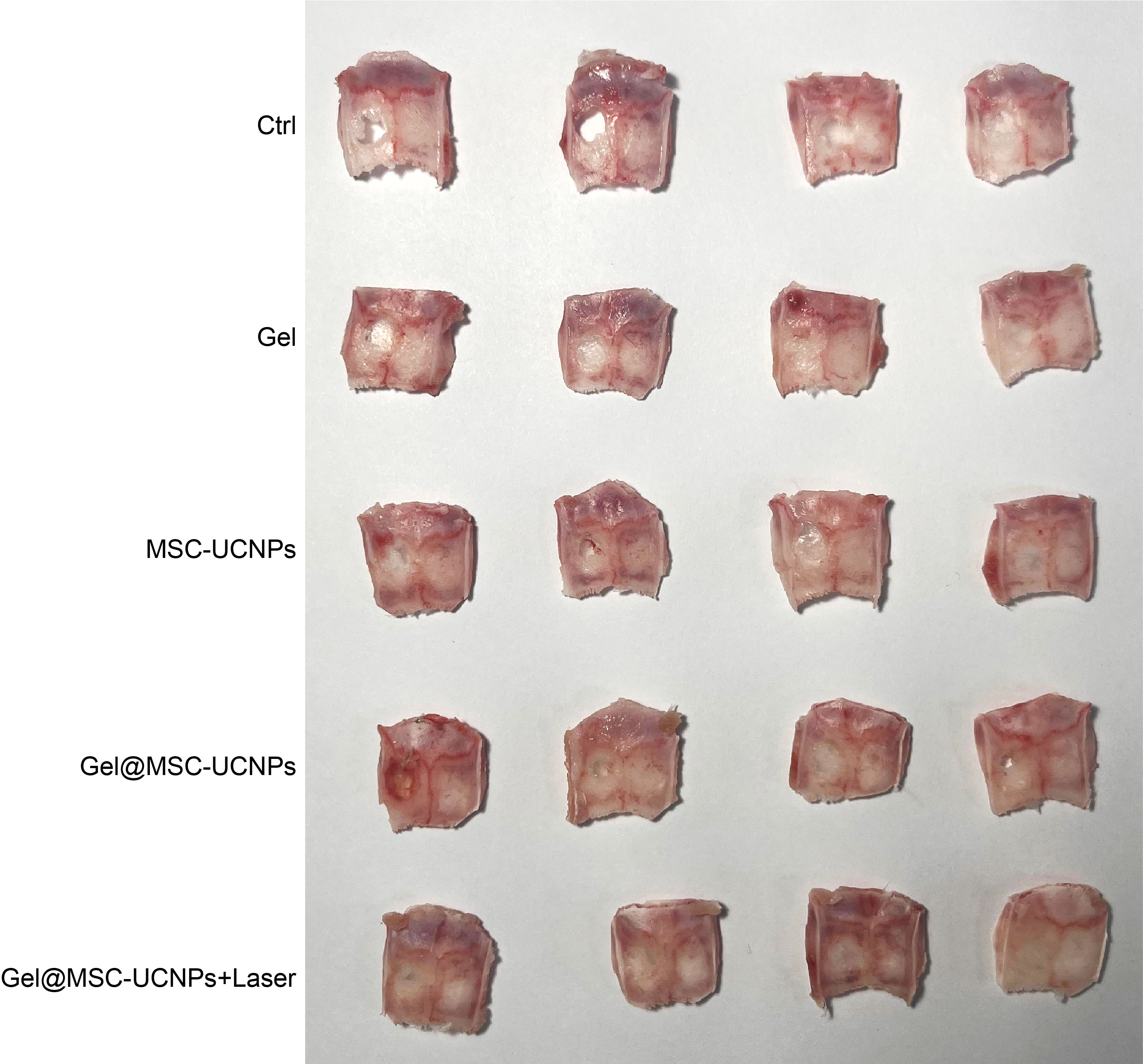


**Figure S6.** Images of isolated calvariae at 8 weeks.


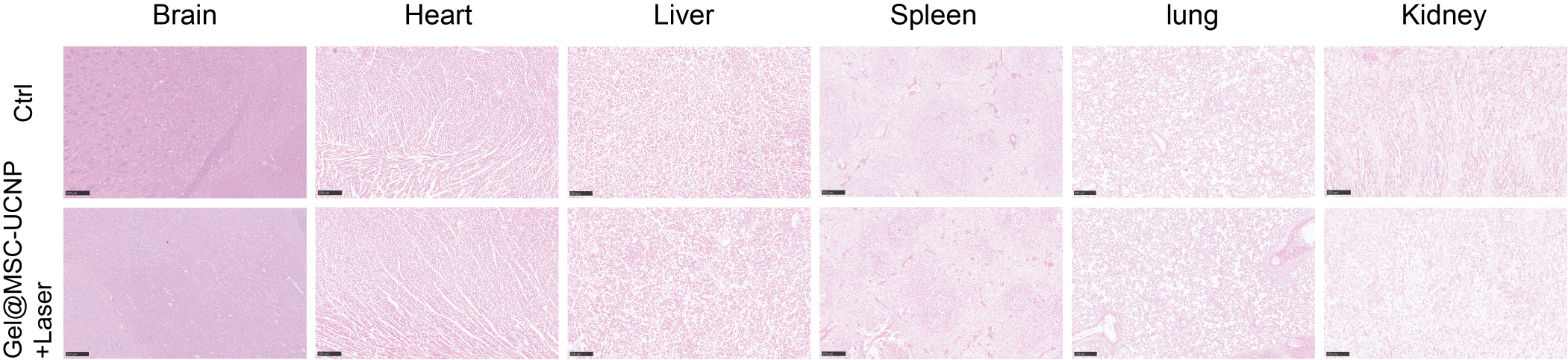


**Figure S7.** Histological analysis using H&E staining of major organs (Scale bar: 250 μm).
